# Engineering the ADDomer Nanoparticle Vaccine Scaffold for Improved Assembly and Enhanced Stability

**DOI:** 10.1021/acssynbio.5c00757

**Published:** 2026-03-17

**Authors:** Georgia Balchin, Burak V. Kabasakal, Alessandro Strofaldi, Sophie Hall, Charlotte Fletcher, Dora Buzas, Joshua C. Bufton, Sathish K. N. Yadav, Dakang Shen, Frederic Garzoni, H. Adrian Bunzel, Jennifer J. McManus, Christiane Schaffitzel, Imre Berger

**Affiliations:** † School of Biochemistry, 1980 University of Bristol, University Walk, Bristol BS8 1TD, U.K.; ‡ Turkish Accelerator and Radiation Laboratory (TARLA), 06830 Ankara, Türkiye; § Department of Biological Sciences, Middle East Technical University, 06800 Ankara, Türkiye; ∥ School of Physics, University of Bristol, Tyndall Avenue, Bristol BS8 1TL, U.K.; ⊥ Department of Chemistry, Maynooth University, Maynooth, Co. Kildare W23 F2H6, Ireland; # King’s College, Lavington Street, London SE1 0NZ, U.K.; ∇ Max Planck Institute for Terrestrial Microbiology, 35043 Marburg, Germany; ○ Max Planck Bristol Centre for Minimal Biology, School of Chemistry,University of Bristol, Cantock’s Close, Bristol BS8 1TS, U.K.

**Keywords:** Adenovirus, Virus-like particles (VLPs), Nanoparticle vaccines, Protein engineering, Thermostability, Electron cryo-microscopy (cryo-EM)

## Abstract

Virus-like particles (VLPs) are promising platforms for next-generation vaccines due to their ability to present antigens in highly ordered, repetitive geometries emulating pathogen-associated patterns to elicit potent immune responses. The ADDomer is a synthetic dodecahedral VLP scaffold derived from the penton base protein (PBP) of human adenovirus serotype 3 (Ad3). PBP tolerates insertion of multiple antigenic epitopes in flexible surface-exposed loops, and spontaneously self-assembles *in vitro* into ADDomer nanoparticles, but faces limitations including incomplete assembly and susceptibility to preexisting antihuman adenovirus immunity. Here, we report two complementary engineering strategies to enhance ADDomer robustness. First, we developed a Chimpanzee adenovirus Y25-based ADDomer (CHIMPSELS) to circumvent preexisting antihuman adenovirus immunity, and introduced a point mutation to restore a motif critical for dodecahedron integrity. Second, we introduced targeted intersubunit disulfide bonds to reinforce particle assembly. High-resolution electron cryo-microscopy confirmed the formation of intact dodecahedral particles, revealing that disulfide bonds stabilize distinct conformations of the PBP N-termini. Differential scanning fluorimetry and dynamic light scattering demonstrated thermal stability and elevated aggregation onset temperatures in the disulfide-stabilized ADDomers, providing a scalable assay for screening ADDomer-based VLP constructs for vaccine development. Incorporation of validated immunogenic epitopes, including a SARS-CoV-2 receptor-binding motif segment and the Chikungunya E2EP3 peptide, demonstrated structural integrity and epitope display by the modified scaffolds. Our results establish a versatile, thermostable VLP platform with reduced susceptibility to preexisting immunity, improved particle integrity, and capacity for modular epitope presentation. This work advances the ADDomer toward practical applications in vaccine development and highlights engineering strategies that can be broadly applied to enhance the performance of protein-based VLP vaccines.

## Graphical abstract

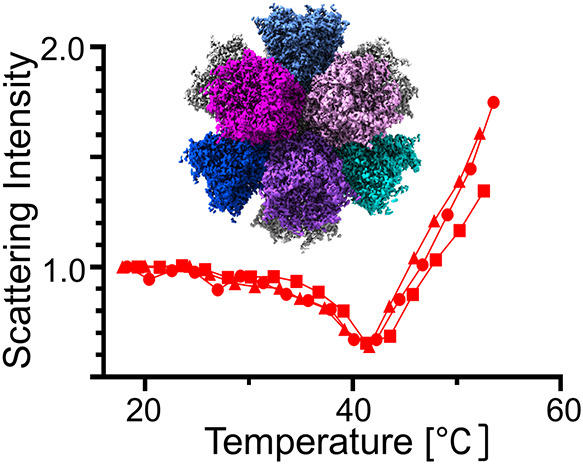

## Introduction

Nanoparticle-based vaccines have emerged as attractive alternatives to traditional vaccine methods. They can be derived from biological or synthetic origins and typically present relevant antigens to the immune system either by surface display or by encapsulation of the antigens.^1^ Among nanoparticle vaccines, virus-like particles (VLPs) represent one of the most advanced classes. VLPs are assembled from one or more proteins, often of viral origin, into particles that mimic the geometry of native viruses, providing a highly repetitive and ordered surface.^2^ This pathogen-associated structural pattern is efficiently recognized by the innate and adaptive immune systems, resulting in strong immunogenicity.^3^ Because VLPs lack genetic material, they are nonreplicative and therefore can offer an advantageous safety profile, even in immunocompromised and elderly populations.^4^ Several VLP based vaccines are already licensed for clinical use, such as the human papilloma virus (HPV) vaccines Gardasil and Cervarix relying on self-assembly of recombinant HPV L1 proteins.^5–7^

Beyond making use of native viral proteins, VLPs can also be engineered to display heterologous epitopes, enabling multivalent presentation of foreign antigens at high density. Such dense, repetitive antigen display promotes efficient B-cell receptor cross-linking and potent antibody responses.^8,9^ In this context, the ADDomer platform we introduced represents a versatile synthetic VLP scaffold.^10^ ADDomer is derived from the penton base protein (PBP) of human adenovirus serotype 3 (Ad3). *In vitro*, 60 PBP subunits spontaneously self-assemble into 12 pentons that further organize into stable dodecahedra, giving rise to a nanoparticle of approximately 30 nm diameter.^10^ The exposed surface of the PBP harbors two flexible loop regions, the so-called variable loop (VL) and arginine-glycine-aspartate (RGD) loop, which tolerate genetic insertions of foreign peptide sequences and even protein domains without disrupting assembly.^10^ This modularity has been exploited to display multiple B- and T-cell epitopes from diverse pathogens, including coronaviruses, foot-and-mouth disease virus, and porcine epidemic diarrhea virus among others, resulting in the generation of specific antibodies following immunization experiments.^11–13^ More recently, engineered ADDomer particles displaying nanobodies against SARS-CoV-2 demonstrated high-avidity binding across multiple viral variants, underscoring their potential as both active and passive immunization tools.^11^

A distinctive feature of ADDomer is its thermotolerance. Thermal shift assays revealed a melting temperature of around 54 °C,^10,11^ moreover, the scaffold tolerates repeated freeze–thaw cycles and prolonged storage at room temperature.^10^ Thermotolerance is highly desirable for vaccine distribution because conventional vaccines often require cold-chain storage at 2–8 °C, or even ultracold conditions of −80 °C, which is costly and logistically challenging, especially in low-resource settings.^14–18^ A vaccine platform such as ADDomer that maintains stability under ambient conditions, could thus significantly reduce distribution costs and improve global accessibility.

Notwithstanding, challenges remain. Electron microscopy has shown that ADDomer preparations often contain a mixture of complete dodecahedra and free pentons, with free pentons constituting up to 30% of the sample, suggesting incomplete assembly, or partial disassembly under the preparation conditions.^10^ Furthermore, preexisting anti-Ad3 adenovirus immunity in humans could potentially affect vaccine performance.^19–21^ One strategy to mitigate this is the use of nonhuman adenovirus-derived scaffolds, such as those based on chimpanzee adenovirus serotypes, which have low seroprevalence in human populations.^19,22^

Protein engineering provides an avenue to enhance VLP stability. In particular, the introduction of disulfide bonds between or within subunits has been shown to increase thermal tolerance in diverse proteins including norovirus-derived nanoparticles and other virus-like assemblies.^23–25^ Rational design guided by high-resolution structural data allows targeted introduction of covalent bonds at intersubunit interfaces, providing predictable and tunable enhancement to particle robustness. Such stabilization strategies can improve particle integrity, reduce disassembly, limit unwanted exposure to non-native epitopes, and improve overall vaccine consistency.

In this study, we report two complementary engineering approaches to improve the robustness of the ADDomer scaffold to address current shortcomings. First, we developed a chimpanzee adenovirus-derived ADDomer to reduce the risk of interference by preexisting immunity to human adenoviruses. Second, we introduced intersubunit disulfide bonds into the PBP to reinforce dodecahedral assembly. Finally, we establish dynamic light scattering (DLS) as a rapid and cost-effective method to assess ADDomer particle stability and integrity, providing a scalable alternative to electron microscopy in screening campaigns to identify most suitable VLP candidates to take forward for a given application.^26^ We demonstrate that these modifications produce highly stable VLPs capable of displaying multiple antigenic epitopes. Together, these approaches expand the versatility of the ADDomer platform, addressing current limitations and advancing its development as a next-generation vaccine scaffold suitable for broad distribution including in remote regions and resource limited settings.

## Results

### Design and Production of CHIMPSELS ADDomer

The original ADDomer VLP was derived from human adenovirus serotype Ad3,^10^ a well characterized and widespread serotype frequently encountered by the immune system of humans. This can result in preimmunity against this adenovirus and components thereof, including the PBP that constitutes the ADDomer protomer. Indeed, *in silico* analyses identified potential immunogenic epitopes in Ad3 PBP.^10^ Therefore, to address limitations that could be caused by preexisting immunity, we developed an ADDomer nanoparticle based on the Chimpanzee adenovirus serotype Y25 (ChAdY25).^19^ ChAdY25 has been used as an adenoviral vector in gene therapy and vaccine applications, notably during the COVID-19 pandemic.^27^ Previously it was found that not all adenoviral PBPs self-assemble into stable nanoparticles *in vitro*, and our first attempts with the ChAdY25 derived PBP confirmed this, with particle assembly seemingly arrested at the penton stage, and no or very little dodecahedra formed (data not shown). Structural studies of ADDomer had revealed a tetrapeptide motif, SELS, within the N-terminal region of the PBP mediating interpenton interactions stabilizing the dodecahedron.^10,28,29^ In the ChAdY25 PBP, however, this motif is altered to SELA (Table S1). We hypothesized that this may be the reason for the lack of stable dodecahedron formation by wild-type ChAdY25 PBP. We thus introduced a single point mutation, A57S, restoring the SELS motif. The resulting construct, CHIMPSELS, was produced using the MultiBac baculovirus/insect cell expression system^30^ and purified to homogeneity by a combination of size exclusion chromatography (SEC) and anion exchange chromatography (AIEX) (Figure S1).

### Cryo-EM Structure of CHIMPSELS ADDomer

The molecular architecture of CHIMPSELS ADDomer VLP was analyzed by cryo-EM at 2.2 Å resolution (Figures S2, S3 and Table S2). The cryo-EM structure showed the expected dodecahedron consisting of 60 PBPs arranged into 12 pentons (Figure 1A,B). In the CHIMPSELS structure, the N-terminus of the PBP comprising the SELS motif including the A57S mutation adopts a hairpin conformation, similar to what we found in the Ad3 ADDomer.^10^ Separate from this study, we have recently determined the cryo-EM structure of a chimeric ADDomer, called CHIMERA. CHIMERA is formed by PBPs based on Ad3, in which the N-terminal part (jelly roll domain) that mediates dodecahedron formation has been replaced with the ChAdY25 jelly roll domain comprising the S57C mutation.^31^ In contrast, the surface exposed part (crown domain) comprising the flexible variable and RGD loops derives from Ad3 (Figure 1C). We noticed that in the CHIMERA VLP, the N-termini adopted a strand-swapped conformation between adjacent pentons (Figures 1C and S4). This is noteworthy, given that the amino acid sequences involved are identical in CHIMPSELS and CHIMERA, but based on our data can adopt two distinct conformations, hairpin or strand-swapped, respectively.

**Figure 1:**
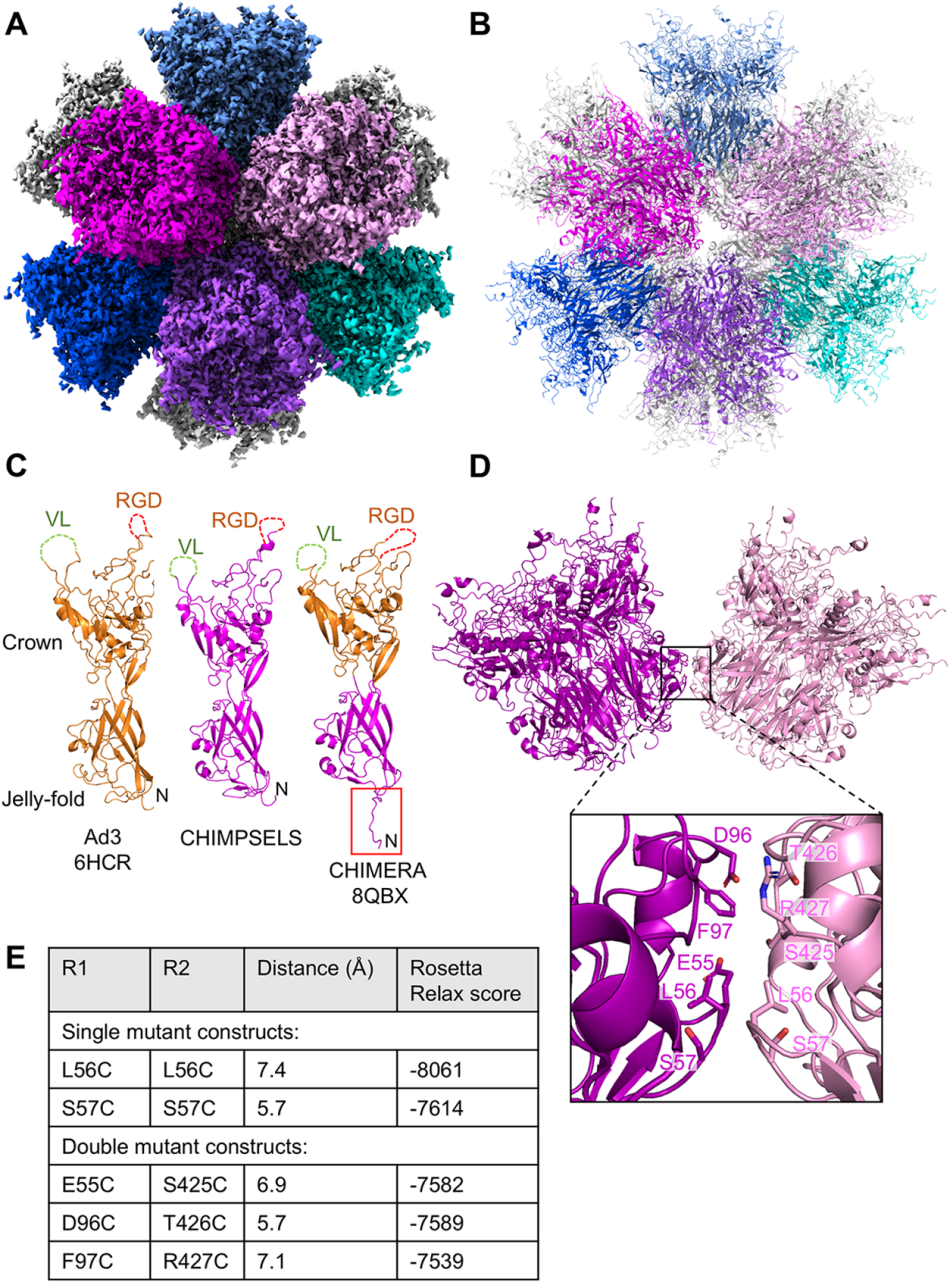
Cryo-EM structure, CHIMPSELS ADDomer. 2.2 Å cryo-EM map (A) and atomic model (B) of the CHIMPSELS ADDomer, comprising 12 pentons shown in different colors. (C) PBP structures of Ad3, CHIMPSELS and CHIMERA ADDomer. Accession numbers are provided. Variable loops (VL) and RGD loops are not resolved in the cryo-EM structures (dashed lines). N-termini are marked. A distinct N-terminal conformation adopted by CHIMERA PBP is boxed in red. (D) Two pentons of CHIMPSELS ADDomer. Zoom-in shows the region of interaction between two PBPs. Residues mutated to cysteine are labeled. (E) The resulting three single and three double mutants, distances between mutated residues, and Rosetta Relax scores.

The pentons in ADDomer VLPs contain a central cavity lined by five glutamate residues, one each per PBP (Figure S5). In the cryo-EM structure of CHIMPSELS, we observed density in the pentons that we attribute to a metal ion. We tentatively assigned this ion as potassium, based on the coordination geometry and distance to the glutamates (3.8 Å).^32^ In a crystal structure of human Ad2 penton, a tightly coordinated Ca^2+^ ion was observed in this position.^29^ In contrast, in CHIMERA and in our previous ADDomer Ad3 cryo-EM structures,^10,31^ no ion is present in the cavity, and the glutamates are rotated away from the central penton axis (Figure S5).

### Disulfide Engineering for Particle Stabilization

We inspected the regions where PBPs from adjacent pentons interact with each other in the available ADDomer cryo-EM structures, with a view to identifying additional interventions at the molecular level that could further stabilize the particle (Figure 1D). This includes the SELS motif identified as key for dodecahedron integrity. Disulfide linkages have been described previously in a number of nanoparticles, enhancing structural integrity.^23–25^ We found residues in the regions we scrutinized that could be mutated to cysteines resulting in geometries conducive to disulfide bond formation in between pentons within the CHIMPSELS ADDomer (Figure 1D). Three double mutants were designed introducing pairs of cysteines within each PBP (E55C S425C; D96C T426C; F97C R427C) to promote two disulfide bonds connecting PBPs in adjacent pentons. Moreover, two single mutants (L56C; S57C) were designed, introducing a single cysteine each in the PBP, aiming to form one disulfide bond linkage at the interface. *In silico* modeling of the resulting disulfide linked pentons using Rosetta software indicated that the single mutants were energetically more favorable as compared to the double mutants (Figure 1E).

All disulfide mutant CHIMPSELS PBPs, as well as CHIMPSELS PBP wild-type and Ad3 PBP, were expressed and purified using the same protocol,^10^ resulting in similar yields. Negative stain EM evidenced dodecahedra, pentons, and amorphous aggregates, depending on the sample analyzed (Figures 2A and S6). Only the two single mutants, CHIMPSELS L56C and CHIMPSELS S57C, formed proper, symmetric dodecahedral particles (Figure 2A). The double cysteine mutants formed larger aggregates or irregularly shaped particles (Figure S6A–C). The micrographs of CHIMPSELS wild-type as well as the L56C and S57C mutants showed very few free pentons as compared to the Ad3 ADDomer (Figure 2A) confirming that the Ad3 ADDomer is significantly less efficient in assembling into dodecahedra, or, alternatively, more prone to breakdown into its constituent pentons, as compared to CHIMPSELS. We also prepared single cysteine mutants (L56C, S57C) and a double mutant (L56C S57C) of the CHIMERA PBP, which has an identical N-terminal region as CHIMPSELS. Negative stain EM evidenced that only CHIMERA S57C formed ADDomer dodecahedra efficiently (Figures 2A and S6D,E) although some of these particles appeared to form higher assemblies in the micrographs. SDS-PAGE analysis confirmed formation of the disulfide bond in the cysteine mutant constructs in the context of the nanoparticles, evidenced by the appearance of a second band migrating at approximately double the molecular weight of the PBP under nonreducing conditions, consistent with disulfide-linked dimers (Figure 2B). However, the PBP monomer band remained more prominent than the PBP dimer band for all mutants tested (Figure 2B). Formation of the disulfide bonds between the cysteine mutant containing PBPs thus appears to remain incomplete, even in the presence of an oxidizing agent (copper phenanthroline) (Figure 2B).

**Figure 2:**
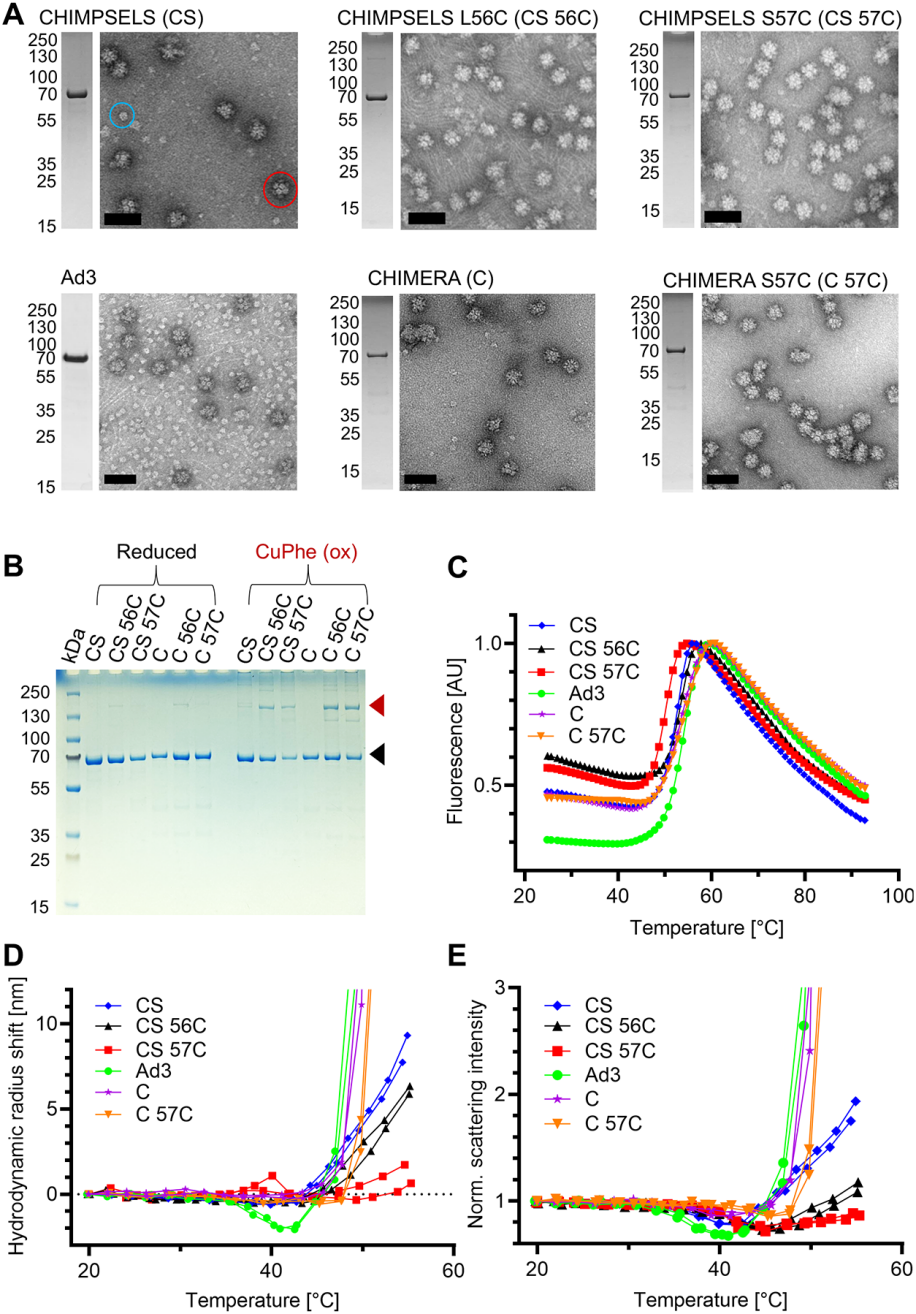
Production and thermostability of CHIMPSELS and CHIMERA cysteine mutant ADDomers. (A) negative stain EM micrographs and SDS-PAGE analysis of CHIMPSELS, Ad3 and CHIMERA wild-type and cysteine mutant ADDomers. A free penton is circled in blue, an ADDomer dodecahedron is circled in red. Scale bars (50 nm) are shown in black. Acronyms used below in panels (B–E) are indicated in brackets. (B) SDS-PAGE analysis of CHIMPSELS, CHIMERA and cysteine mutants under reducing and oxidizing conditions. Black arrow indicates PBP monomers, red arrow indicates disulfide linked PBP dimers. CuPhe, copper phenanthroline. (C) Thermofluor analysis of CHIMPSELS, CHIMERA and cysteine mutant ADDomers. (D) Hydrodynamic radius shifts by DLS. (E) Normalized scattering intensities. Two plots are shown for each construct.

### Thermotolerance and Onset of Aggregation

The thermotolerance of wild-type and cysteine mutant ADDomer nanoparticles was investigated by differential scanning fluorimetry (DSF) to identify melting temperatures (*T*_m_) (Figure 2C and Table S3). All ADDomer particles had melting temperatures exceeding 50 °C. We measured the highest *T*_m_ (54.3 °C) in our experiments for Ad3 ADDomer while the cysteine mutant CHIMPSELS S57C exhibited the lowest *T*_m_ (50.2 °C). Dynamic light scattering (DLS) was used to analyze the size of the nanoparticles at increasing temperatures in the range of 25 to 55 °C. We observed that from 25 to 40 °C, particle size did not change significantly, but at temperatures above 40 °C, the average hydrodynamic radius of all ADDomer samples increased, in some cases markedly (Figure 2D). This increase in hydrodynamic radius was coupled to an increase in scattering intensity at temperatures between 45 and 55 °C (Figure 2E), confirming that samples were aggregating in this temperature range.^33^ However, we also observed a small decrease in scattering intensity for all ADDomer samples before the described increase occurred, at around 35 to 45 °C, an observation linked to protein unfolding^33^ or swelling in previous studies of viral capsids of Southern bean mosaic virus and adenovirus Ad2.^34,35^ While proper VLPs swelling would be indicated by simultaneous increase in hydrodynamic radius and decrease in scattering intensity, CHIMPSELS and CHIMERA constructs displayed constant hydrodynamic radii prior to aggregation. Given that protein unfolding between 35 and 45 °C was ruled out by DSF experiments, we speculate this phenomenon may be due to particle density variations caused, for instance, by water permeation, or small amounts of VLP disassembly, which was observed by negative stain EM for CHIMPSELS constructs at 45 °C (Figure S7). This is not observed by DLS as the larger VLP particles more strongly scatter light.^26^ On the contrary, the Ad3 ADDomer was the only construct showing concomitant hydrodynamic radius and scattering intensity decrease (Figure 2D); hence, this is likely due to the disassembly of ADDomer dodecahedrons into pentons which was also observed by negative stain EM (Figures 2A and S7). In fact, in the micrographs of the samples analyzed, we observed some pentons emerging at higher temperatures, which is not readily observed by DLS.^26^

The Ad3, CHIMERA and CHIMERA S57C ADDomers aggregated at a markedly faster rate at high temperature showing a steep increase in hydrodynamic radius and scattering intensity by DLS, as compared to the ADDomers based on the CHIMPSELS scaffold (Figure 2D,E). Ad3, CHIMERA and CHIMERA S57C share identical amino acid sequences in their crown domains.^10,31^ It is thus likely that this region is responsible for this pronounced aggregation, perhaps following partial disassembly of the ADDomer particles.

For both CHIMPSELS and CHIMERA, introduction of disulfide bonds markedly shifted the temperature of the onset of aggregation, consistent with the mutant particles being significantly more resistant to aggregation at higher temperatures. CHIMPSELS S57C exhibited an aggregation onset temperature of 49 °C, compared to 44.5 °C of wild-type CHIMPSELS (Figure 2D,E). Moreover, the hydrodynamic radius shift, as well as the scattering intensity increased only modestly up to 55 °C (Figure 2D,E). This is consistent with the negative stain EM micrographs which also show much less aggregation of CHIMPSELS S57C at 50 °C as compared to wild-type (Figure S7). The temperature at which the scattering intensity decreased was also higher for CHIMPSELS S57C as compared to wild-type CHIMPSELS, suggesting that any subtle particle instability, if present, happens only at a higher temperature when the disulfide linkage is present (Figure 2E). Taken together, our results suggest that CHIMPSELS S57C is superior to the other scaffolds analyzed, in terms of structural integrity, resistance to deformation and a delayed onset of aggregation, while maintaining a *T*_m_ close to that of the wild-type CHIMPSELS, CHIMERA and Ad3 scaffolds.

### Cryo-EM Structure of CHIMPSELS S57C ADDomer

To better understand the effects of the cysteine mutation on ADDomer particle integrity, CHIMPSELS S57C ADDomer was purified to homogeneity (Figure S8) and analyzed by cryo-EM at 2.14 Å resolution (Figures 3 and S9). Initially, the N-terminal region comprising the S57C mutation was not unambiguously resolved. Therefore, masked 3D classification was applied to two adjacent pentons comprising the interacting PBPs (Figure 3B), to a resolution of 2.61 Å (Figure S10). Interestingly, we observed both a strand-swapped and a hairpin conformation adapted by the N-termini in the resulting refined EM density (Figure 3C). Moreover, it appears that the covalent linkage can only be formed efficiently in the hairpin conformation that juxtaposes the cysteines of the interacting PBPs in a geometry compatible with disulfide bond formation (Figure 3C). This could explain the incomplete PBP dimer formation observed in SDS-PAGE, even if an oxidizing agent was added (Figure 2B).

**Figure 3:**
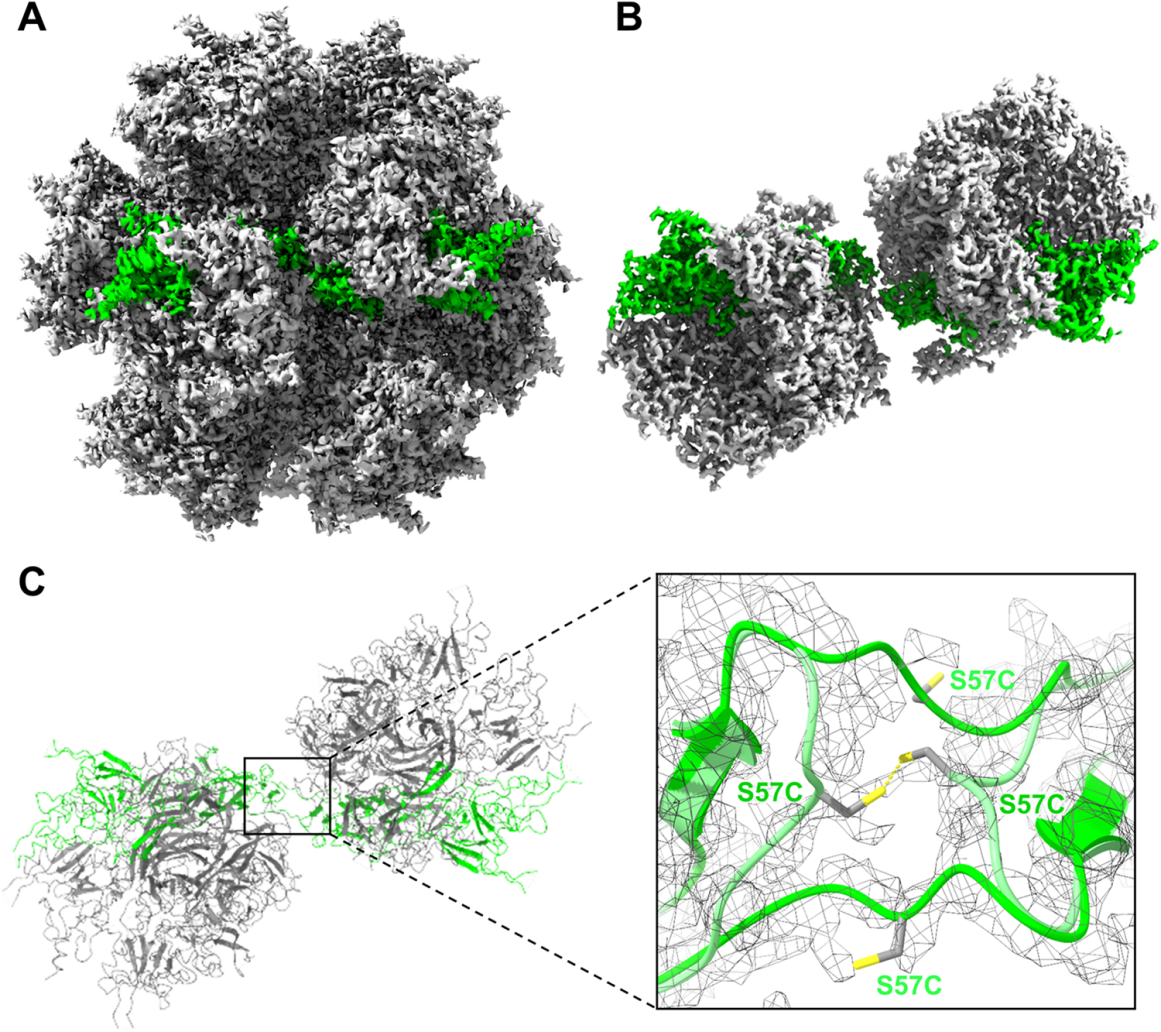
Cryo-EM structure, CHIMPSELS S57C ADDomer. (A) 2.1 Å cryo-EM map of CHIMPSELS S57C ADDomer. Two interacting PBPs in adjacent pentons are highlighted in green. (B) The two interacting pentons are shown in a cut-out. (C) Interacting region in a zoom-in with the corresponding cryo-EM density shown (gray mesh). Two alternate conformations of the N-termini are observed, a hairpin (light green) and a strand-swapped conformation (bright green). Cysteine residues (gray) introduced in the mutant were modeled in the reduced form. Sulfur atoms are colored yellow.

### Epitope Insertion for ADDomer VLP Vaccine Candidates

The receptor binding motif (RBM) of SARS-CoV-2, the virus causing COVID-19, is a well-characterized immunogenic antigen recognized by antibodies.^36^ The E2EP3 peptide from the Chikungunya E2 glycoprotein is the major neutralizing epitope found in the sera of infected patients.^37^ We had utilized both epitopes previously to design Ad3 ADDomer-based vaccine candidates.^10,13^ Epitopes comprising a segment of SARS-CoV-2 RBM (CoV), or the complete Chikungunya E2EP3 peptide (Chik), respectively, were inserted into the VL of CHIMPSELS S57C, to validate the use of this engineered scaffold toward improved thermostable VLP vaccine candidates against infectious diseases, resulting in CHIMPSELS S57C ADDomer CoV and CHIMPSELS S57C ADDomer Chik (Figure 4). As could be expected, in both cases, epitope insertion (Figure 4A) was compatible with disulfide bond formation resulting in PBP dimers and even additional multimers in denaturing, nonreducing SDS-PAGE (Figure, 4B). The E2EP3 epitope is located at the extreme N-terminus of the E2 glycoprotein in the Chikungunya virus.^37^ Thus, to liberate the E2EP3 epitope N-terminus in the CHIMPSELS S57C ADDomer Chik, a Tobacco etch virus (TEV) protease cleavage site was placed into the VL loop, immediately before the E2EP3 epitope, and the purified ADDomer was treated with TEV to realize a more native-like E2EP3 epitope presentation^10^ (Figure 4B). When the CHIMPSELS S57C ADDomer Chik sample was treated with TEV protease, the PBP band at 70 kDa became much less prominent, and two bands appeared, indicating the PBP was properly cleaved into polypeptides of approximately 18 kDa and 45 kDa molecular weight (Figure 4B). Addition of oxidizing agent led to complete disappearance in SDS-PAGE of the 18 kDa band, which contains the S57C residue, and a band appeared at approximately 40 kDa consistent with a disulfide-linked dimer. Negative stain EM confirmed the integrity of the CHIMPSELS S57C VLP vaccine candidates evidencing dodecahedra, with virtually no pentons or aggregates present (Figure 4C–E). The micrographs of CHIMPSELS S57C ADDomer Chik before and after TEV cleavage (Figure 4D,E) evidenced that introduction of up to 60 cuts into the PBP polypeptide chains did not noticeably affect the integrity of the nanoparticle.

**Figure 4:**
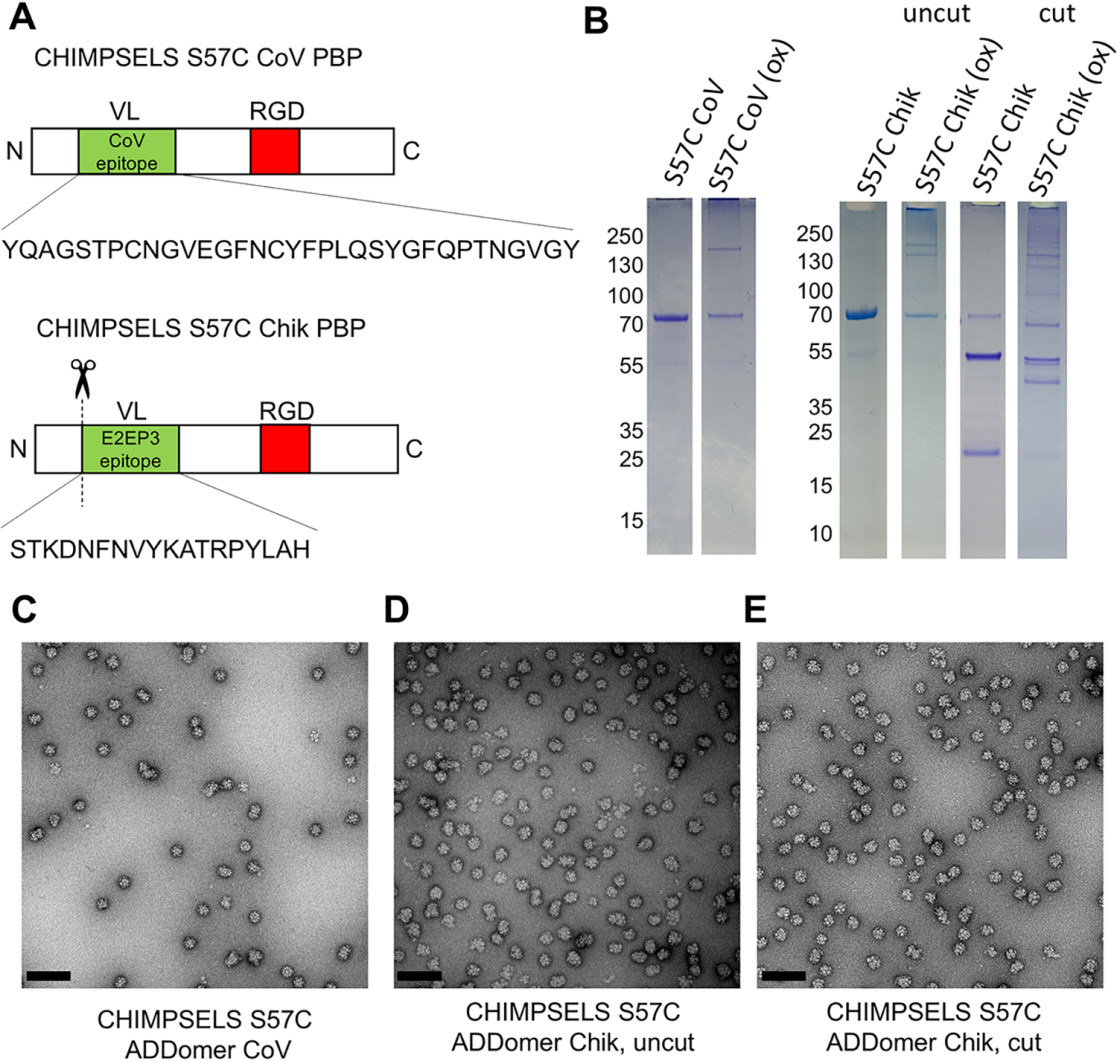
CHIMPSELS S57C ADDomer vaccine candidates. (A) Schematic of CHIMPSELS S57C CoV PBP with CoV epitope (sequence below) inserted into VL (green). RGD loop is shown in red. (B) Schematic of CHIMPSELS S57C Chik PBP with E2EP3 epitope (sequence below) inserted into VL. Scissors and dashed line indicate TEV cleavage to expose the E2EP3 N-terminus in a native like conformation. (C) SDS-PAGE analysis of CHIMPSELS S57C ADDomer CoV in reducing and oxidizing conditions. (D) CHIMPSELS S57C ADDomer Chik in reducing and oxidizing conditions, before and after TEV cleavage. (E–G) Negative stain EM of CHIMPSELS S57C ADDomer CoV, and CHIMPSELS S57C ADDomer Chik before and after treatment with TEV protease. Scale bar (100 nm) is shown in black.

Next, we carried out DLS and thermal shift experiments, to analyze the effect of 60 antigenic epitopes displayed on the ADDomer surface, on aggregation and thermotolerance (Figure 5). DLS was carried out for CHIMPSELS S57C ADDomer CoV following the protocol used for CHIMPSELS, except for the addition of 200 mM sodium iodide (NaI) into the buffer. Hydrodynamic radii and scattering intensities were recorded (Figure 5A,B). The DLS results followed the same pattern as we saw with the CHIMPSELS S57C scaffold. We observed a decrease in scattering intensity at around 35 °C which appears more pronounced with the CoV epitopes inserted. Aggregation onset occurred at about 44 °C, as compared to 49 °C for the scaffold, suggesting that the addition of the RBM derived epitopes makes the particle more prone to aggregation at lower temperatures than the unmodified variant.

**Figure 5:**
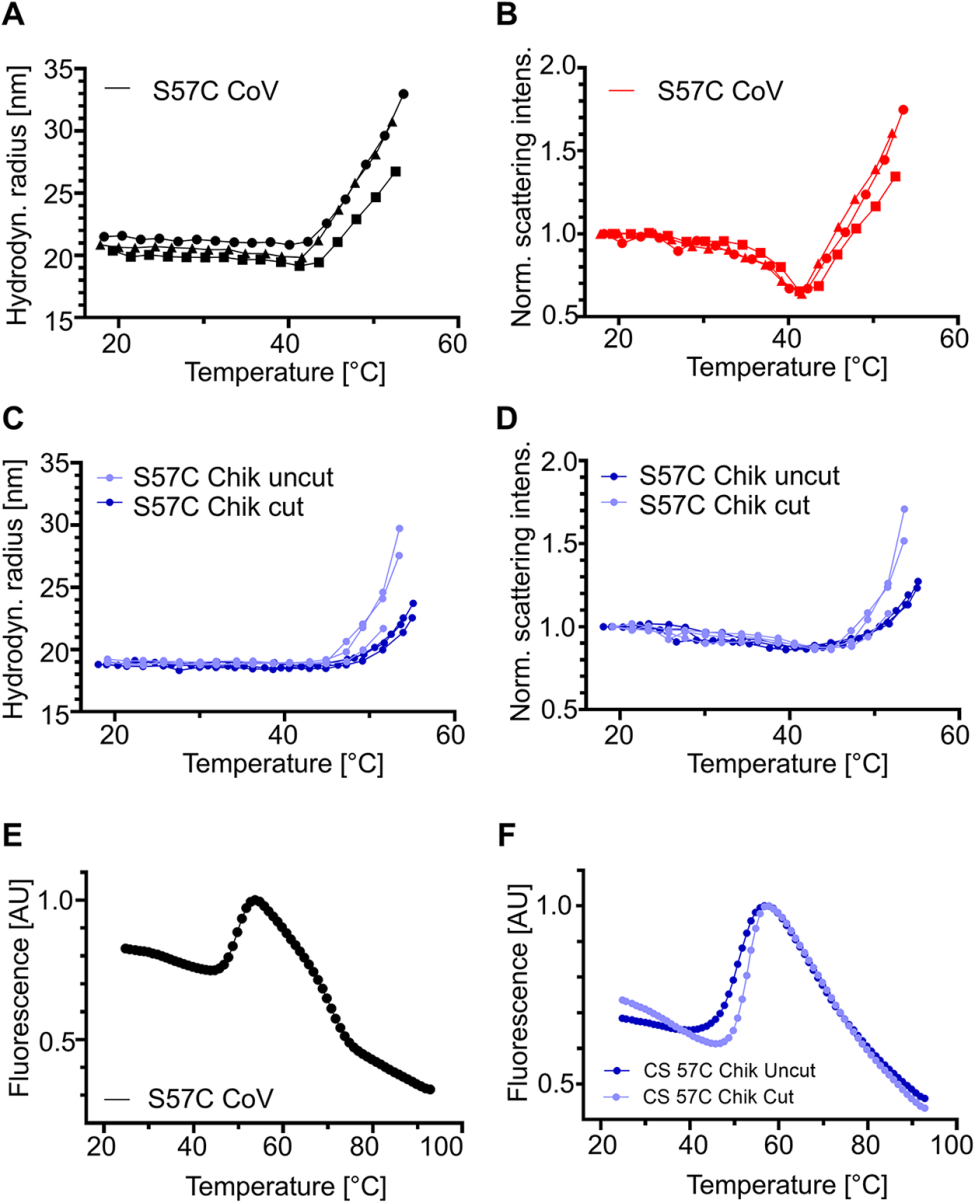
Thermostability screen, CHIMPSELS S57C ADDomer VLP vaccine candidates. Hydrodynamic radius from DLS (A) and scattering intensity (B) of CHIMPSELS S57C ADDomer CoV, in triplicate. Hydrodynamic radius from DLS (C) and scattering intensity (D) of CHIMPSELS S57C ADDomer Chik cut with TEV protease (light blue) and uncut (dark blue), in triplicate. (E) Thermofluor analysis of CHIMPSELS S57C ADDomer CoV. (F) Thermofluor analysis of the CHIMPSELS S57C ADDomer Chik cut with TEV protease (light blue) and uncut (dark blue). S57C Chik, CHIMPSELS S57C ADDomer Chik VLP vaccine candidate.

DLS of CHIMPSELS S57C ADDomer Chik, uncut and cut with TEV protease showed identical hydrodynamic radii, indicating that TEV cleavage did not affect particle size (Figure, 5C). Both samples followed the same trajectory as CHIMPSELS S57C with the hydrodynamic radii and scattering intensities unchanged up to 40 °C, but increasing at higher temperatures, indicating aggregation (Figure 5C,D). This occurred at virtually identical temperatures for TEV cleaved VLP (47 °C) and uncut sample (48 °C), as compared to the CHIMPSELS S57C scaffold (49 °C). A small dip could be observed in the scattering intensity of both cut and uncut samples, from around 35 to 45 °C (Figure 5D), although this was less pronounced than what we observed for CHIMPSELS S57C ADDomer CoV.

Thermofluor experiments were carried out to assess the thermotolerance of the vaccine candidates, showing a *T*_m_ of about 50 °C for CHIMPSELS S57C ADDomer CoV (Figure 5E). However, the starting fluorescence was rather high resulting in a relatively small assay window for the values measured during the transition from folded to unfolded states. Elevated starting fluorescence indicates that the protein is hydrophobic and the dye used (SYPRO orange) may bind to hydrophobic patches exposed on the surface of the protein, prior to unfolding.^38^ Thermal shift assays of CHIMPSELS S57C ADDomer Chik, uncut and cut with TEV protease, yielded *T*_m_ values of 51.6 and 53.3 °C, respectively, which were higher than the *T*_m_ of CHIMPSELS S57C without epitopes (50.2 °C). Of note, the *T*_m_ of uncut VLP was lower than the *T*_m_ of the TEV cut sample, suggesting unfolding occurs at a higher temperature when the polypeptide chains of the 60 PBPs that constitute the VLP are cleaved in their loop regions.

## Discussion

In this study, we engineered a synthetic self-assembling dodecahedral nanoparticle based on the PBP of a chimpanzee adenovirus, ChAdY25, to advance the ADDomer platform toward practical vaccine applications. One reason for moving away from the human adenovirus type 3 (Ad3) scaffold previously implemented by us and others as a VLP platform for vaccine candidates against a range of human and animal diseases,^10–13^ was to mitigate the risk of interference by preexisting immunity, which can limit the effectiveness of adenovirus-based vectors in humans.^20,21^ Although the ChAdY25 serotype has now been widely deployed as a replication-deficient vector in COVID-19 vaccines,^22,27^ the majority of scaffold antibodies that are elicited target the adenoviral hexon protein. Because our scaffold is composed solely of penton proteins, it does not present hexon epitopes and is therefore less susceptible to interference from antihexon immunity.^10,22^ Thus, the CHIMPSELS ADDomer represents a viable scaffold with reduced susceptibility to neutralization by preexisting antibodies. We envision that CHIMPSELS may be used broadly, also in combination with diverse ADDomers derived from rare serotypes, in future vaccine campaigns against multiple infectious diseases.

Structural studies by EM revealed that CHIMPSELS ADDomer forms stable dodecahedra, and that the engineered restoration of the SELS motif is critical for assembly and integrity of this VLP. Interestingly, the N-terminal region of PBPs was observed to adopt alternative conformations, hairpin or strand-swapped, in different ADDomers, in spite of identical amino acid sequence. It is not entirely clear currently from the available structures why the same sequence adopts distinct geometries in different VLPs. One explanation could be that long-range allosteric effects originating from the different crown domains within the PBP may influence the conformation of the N-terminus. However, identical hairpin conformations observed in CHIMPSELS and Ad3 ADDomers in spite of different crown domains, appear to challenge this view. Alternatively, intracellular production yields could impact on the folding pathways in this region, and intermolecular strand-swapping may be preferred to intramolecular hairpin formation at very high recombinant expression levels. These observations broaden our understanding of adenovirus-derived nanoparticle assembly and highlights the importance of subtle sequence–structure relationships in particle stability.

To further enhance robustness, we applied disulfide engineering at interpenton interfaces. Consistent with previous work on unrelated VLPs,^23–25^ single-cysteine variants of the PBP were capable of forming stable ADDomer particles, whereas double-cysteine mutants appeared to misassemble, possibly due to mispaired linkages. Even though disulfide bond formation was incomplete, structural, and biophysical analyses demonstrated that the S57C mutation improved assembly efficiency, reduced the presence of free pentons, and delayed the onset of aggregation at elevated temperatures. Cryo-EM analysis of the CHIMPSELS S57C VLP evidenced coexistence of the hairpin and strand-swapped conformations of the PBP N-termini. Based on the molecular geometries revealed by cryo-EM, disulfide bond formation occurs preferentially when the PBP N-terminus adopts the hairpin conformation, offering a structural explanation why disulfide bond linkage of the PBPs did not reach completion even in the presence of an oxidizing agent. Collectively, these findings establish a framework for stabilizing VLPs through targeted covalent engineering while underscoring the balance between stabilization and conformational flexibility.

Thermal stability is a central requirement for next-generation vaccine platforms, particularly in settings where cold-chain infrastructure is limited.^14–18^ Our data indicate that CHIMPSELS S57C ADDomer resists aggregation more effectively than Ad3- and CHIMERA-based scaffolds, while maintaining a comparable thermal melting temperature. This suggests that disulfide reinforcement strengthens particle integrity without compromising intrinsic thermotolerance. Such stability is especially relevant for global vaccine distribution, where thermostable VLPs could significantly reduce costs and logistical barriers.

Importantly, the engineered scaffold tolerated insertion of diverse viral epitopes, including a SARS-CoV-2 RBM derived epitope and the Chikungunya E2EP3 peptide, without loss of assembly, stability, or disulfide bond formation propensity. The structural integrity of these vaccine candidates was preserved, including after proteolytic processing of the E2EP3 peptide-containing VLPs, demonstrating that the engineered ADDomer can accommodate complex epitope designs and tolerate proteolytic cleavage of each PBP polypeptide chain in a surface exposed region. While insertion of hydrophobic epitopes modestly reduced the aggregation onset temperature, this effect could be mitigated by buffer optimization, suggesting that formulation strategies can further enhance stability.

Taken together, our work introduces two complementary strategies, serotype substitution and disulfide engineering, that overcome current limitations of the ADDomer scaffold technology. The CHIMPSELS S57C VLP platform provides improved assembly efficiency, reduced risk of preexisting immunity, and enhanced resistance to aggregation, while maintaining compatibility with multivalent epitope display. Nonetheless, incomplete disulfide bond formation remains, and waits to be addressed by further engineering. Moreover, the immunogenicity of the vaccine candidates will need to be validated *in vivo*. Future work should thus focus on expanding the structural design space for covalent stabilization, systematic assessment of epitope-dependent effects on stability, and evaluation of immune responses in relevant animal models. In the present work, we have established the molecular foundation and an assay system to analyze and address these aspects at scale.

In conclusion, this study establishes a versatile and thermostable nonhuman adenovirus derived ADDomer scaffold with reduced susceptibility to preexisting adenoviral immunity. By combining structural engineering with epitope modularity, we provide a broadly applicable strategy for advancing protein-based nanoparticle vaccines toward clinical utility.

## Materials and Methods

### Production of ADDomers

Sequences encoding ADDomer PBPs described in this study were inserted into pACEBac1 plasmids (Geneva Biotech SARL, Switzerland) and expressed using the MultiBac baculovirus/insect cell system.^30^ *Spodoptera frugiperda* Sf21 cells were used for virus generation and amplification, and *Trichoplusia ni* Hi5 cells for ADDomer VLP production. All ADDomers were expressed at 27 °C except for the ADDomer VLP vaccine candidates, which required expression at 19 °C. In this case, Hi5 cells were cultured at 27 °C, infected with the respective recombinant baculovirus, and subsequently shifted to 19 °C for protein expression.

ADDomer VLPs were purified following established protocols.^10,28,39^ After purification, the ADDomer samples to be studied by DLS were buffer exchanged into a No-salt Buffer 50 mM Tris, 0.2 mM EDTA, 0.1 mM AEBSF (4-(2-Aminoethyl)­benzenesulfonyl fluoride, pH7.4) using 100 kDa Amicon Ultra centrifugal filter units (Millipore). ADDomer-based Chikungunya vaccine candidates presenting the E2EP3 epitope were cleaved by TEV protease by adding 20 U of TEV protease per μg ADDomer, followed by incubation at 4 °C overnight with constant rotation. The hexa-histidine tagged TEV protease was removed using HisPur cobalt resin (Thermo Scientific) as follows: 1 mL of resin slurry was washed with 5 mL Milli-Q water for 5 min and equilibrated with 5 mL buffer, then incubated with TEV cleaved ADDomer sample for 1 h at 4 °C. Removal of TEV protease was confirmed by SDS-PAGE and Western blot analysis.

### Negative Stain EM

Negative stain EM was performed at 0.1 mg/mL sample concentration. CF300-Cu grids (Electron Microscopy Sciences) were glow discharged at 40 mA for 30 s, 5 μL purified sample was applied to the grid and incubated for 1 min, then manually blotted onto filter paper. The grid was then washed with 5 μL 3% uranyl acetate, blotted, and 5 μL 3% uranyl acetate applied to the grid followed by incubation for 1 min, before blotting and washing the grid a final time with 5 μL of 3% uranyl acetate. Grids were imaged at 49,000× magnification on a FEI Tecnai 12 120 kV BioTwin Spirit microscope (Thermo Fisher) with an Eagle 4k × 4k CCD camera.

### Cryo-EM Structure Analysis of CHIMPSELS ADDomer

Data collection: Purified CHIMPSELS ADDomer was applied to a glow discharged holey carbon grid (Quantifoil, R 2/2 300 mesh) and blotted before plunge freezing in liquid ethane using a Vitrobot (Thermo Fisher). Cryo-EM micrographs were acquired using an FEI Talos Arctica microscope equipped with a Gatan K2 detector operating in super-resolution mode with an energy filter. A total of 2920 dose-fractionated movies were recorded, each comprising 40 frames with an exposure time of 0.2 s per frame. The total electron dose was 44.01 e^–^/Å^2^, corresponding to a pixel size of 1.05 Å. Data collection was performed at a nominal magnification of 130,000×, with a defocus values ranging from −0.7 μm to −2.2 μm, incremented in 0.5 μm steps.

Data processing: Cryo-EM data was processed using the Relion 3.1 software package.^40,41^ First, motion correction of micrographs was performed with MotionCorr2,^42^ and contrast transfer function (CTF) was estimated using CTFfind.^43^ Micrographs with a resolution of better than 3.7 Å were selected for particle picking (2077 out of 2920). After two-dimensional (2D) classification, and 3D classification with no symmetry imposed, 147098 particles were used for the initial 3D refinement with imposed icosahedral (I4) symmetry, resulting in a map at 2.6 Å resolution. After CTF and aberration refinement, and Bayesian polishing, the final postprocessed map reached up to 2.2 Å based on the Fourier shell correlation (FSC) 0.143 cutoff.^44^ Local resolution of the final map was determined in Relion 3.1 and visualized with ChimeraX.^45^

Model building: The initial model was generated *ab initio* with the EM map and amino acid sequence using Buccaneer^46^ in the CCPEM suite.^47^ The model was completed and built manually with Coot^8^ by applying noncrystallographic symmetry from the crystal structure of the human adenovirus Ad2 penton.^29^ The model refinement was performed by real-space in Phenix^49^ iteratively, followed by model building in Coot. The final structure was validated using MolProbity.^50^

### Structure-Based Cysteine Mutant Construct Design

Based on the structure of CHIMPSELS ADDomer, five cysteine mutants were designed. First the unresolved VL and RGD loop were modeled using Rosetta.^51–55^ To that end, 14 symmetrical penton models were constructed with Rosetta SymDock,^53,54^ maintaining the 5-fold symmetry during all subsequent steps. The missing loops were modeled in a stepwise process using Rosetta Remodel^51,55^ based on the highest scoring model, resulting in 14 models. The best scoring loops were further relaxed using Rosetta Relax,^52^ resulting in 140 models. The best scoring relaxed model was subsequently relaxed into the 60mer dodecahedron particle by aligning the pentons to the CHIMPSELS ADDomer cryo-EM structure, and running the Rosetta Relax program while imposing 60-fold symmetry.

For designing the cysteine mutant ADDomers, two adjacent penton models were extracted from the CHIMPSELS ADDomer structure. Cysteine mutations were introduced in the models using PyMol (Schrödinger LLC) only for the chains that form the penton dimer interface, and the resulting models were relaxed 10 times using Rosetta Relax. The final scores correspond to the average of the resulting total scores of these models. Cysteine mutant ADDomers were also prepared using the CHIMERA ADDomer particle.^31^ Single cysteine mutants S57C and L56C that resulted in properly formed CHIMPSELS ADDomer particles were introduced also in CHIMERA, which contains a jelly roll domain with an identical amino acid sequence. A double mutant was also prepared in the same way, including both the L56C and S57C mutation.

### Cryo-EM of CHIMPSELS S57C ADDomer

Data collection: 4 μL of 0.5 mg/mL CHIMPSELS S57C ADDomer sample was applied to a holey QUANTIFOIL R 1.2/1.3 grid with an ultrathin carbon film (Sigma-Aldrich) that was glow discharged in air with a current of 4 mA for 120 s. The grid was blotted for 2 s at 4 °C with 100% relative humidity before plunge-freezing in liquid ethane-propane. Micrographs were collected using a 300 kV FEI Titan Krios microscope (Thermo Scientific) equipped with a Gatan K3 detector and an energy filter. A total of 20,080 dose-fractionated movies were collected with 50 frames each using a total exposure time of 3.846 s. The total electron dose was 50 e^–^/Å^2^, corresponding to a pixel size of 1.072 Å. Data collection was performed at a nominal magnification of 81,000× and a defocus range of −0.8 μm to −2 μm with incremental steps of 0.4 μm.

Data processing: Image processing was performed as described above for CHIMPSELS ADDomer. From 16,511 micrographs, 2,464,479 particles were boxed using the RELION 4.0 auto picking software. Particles were subjected to 2D classification resulting in 1,442,068 particles. Rounds of 3D classification were performed using the CHIMPSELS ADDomer structure as a reference low pass filtered to 60 Å. Particle polishing was performed, and the map was refined using the RELION 4.0 3D refinement tool and the postprocessing tool with automatic B-factor sharpening using a B-factor value of −60.7 resulting in a map with a resolution of 2.7 Å (Gold standard FSC 0.143 criterion).^44^ Icosahedral (I4) symmetry was also imposed, and postprocessing was performed with a B-factor value of −73.2 resulting in a map with 2.14 Å resolution (Gold standard FSC 0.143 criterion).^44^

A map of two adjacent pentons was created using UCSF ChimeraX software^56^ and this was used to create a map covering two pentons for masked 3D classification. The two best of six classes were refined using RELION 3D refine, resulting in maps with 2.97 Å and 4.0 Å resolution. The 2.97 Å map was postprocessed using the RELION tool applying a B-factor value of −52.4 resulting in a resolution of 2.64 Å.

Model building: The CHIMPSELS ADDomer model was used as a starting point for model building and refinement. Two pentons were extracted from this model and fit into the two-penton cryo-EM density map using UCSF ChimeraX,^45^ and the S57C mutations was introduced. Subsequent rounds of refinement were carried out using the real-space refinement tool in the Phenix^49^ and Coot.^48^ The model was evaluated using Phenix Real-space refine, and EMRinger.^56^

### Reduced/Oxidised SDS-PAGE

100 μM of copper phenanthroline was added to respective ADDomer VLP samples at 10 μM, incubated at 4 °C for 1h, followed by dilution to 0.1 mg/mL. Each sample was split in two, and reducing protein gel loading buffer (PGLB) was added to one-half, while the other half was supplemented with nonreducing PGLB. Both samples were heated to 96 °C for 5 min using a heat block before analysis by SDS-PAGE.

### Dynamic Light Scattering (DLS)

ADDomer samples at 0.2 mg/mL in No-salt Buffer were centrifuged at 21,300*g* for 30 min at 4 °C. For CHIMPSELS S57C ADDomer CoV samples, 200 mM NaI was supplemented to the buffer. The supernatant was removed and double filtered through 0.22 μm Millex PDVF Syringe Driven Filter Units (Millipore). At least 180 μL was placed into a 50 mm glass tube (Hilgenberg GmbH) and sealed with PTFE tape. The DC30-K20 external circulating water bath (Thermo Scientific) controlling the temperature of the DLS instrument was set to approximately 20 °C and the sample was allowed to equilibrate in the instrument for 5 min prior to measurement. DLS measurements were recorded using an ALV/CGS-3 goniometer system with a HeNe laser (632.8 nm wavelength) and an optical fiber-based detector along with an ALV/LSE-5004 digital correlator. DLS measurements were taken using the quickset mode so both DLS and static light scattering (SLS) could be recorded simultaneously at a 90° scattering angle. A dust filter was applied. Temperature ramp experiments from 20 to 55 °C were performed with measurements taken at increments of 2 to 2.5 °C, with the temperature controlled by the water bath. For each temperature point the cumulant fit of the correlation function was used to give the first order hydrodynamic radius, polydispersity index (PDI) and scattering intensity. The viscosity and refractive index were corrected for each temperature. Hydrodynamic radius, change in hydrodynamic radius and normalized scattering intensities were plotted against temperature as described.^57^ Experiments were carried out in triplicate unless indicated otherwise.

### Thermal Shift Assay (Thermofluor)

Thermal shift assays were carried out using a customized version of a previous protocol.^58^ In our experiments, 25 μL reactions were set up on a MicroAmp Optical 96-Well Reaction Plate (Applied Biosystems) containing 5× SYPRO Orange Protein Gel Stain (Invitrogen) and 1 mg/mL ADDomer sample concentration in No-salt Buffer (supplemented with 200 mM NaI in case of CHIMPSELS S57C ADDomer CoV). For ADDomer samples containing an engineered disulfide bond, copper phenanthroline was added to 100 μM. A temperature ramp from 25 to 95 °C at an interval of 1 °C/min was applied using a Mx3005P real-time PCR system. At every 1 °C step, the fluorescence intensity of the SYPRO Orange dye was measured using excitation and emission wavelengths of 492 and 516 nm, respectively. The raw fluorescence intensity was normalized and plotted against temperature. The thermal melting temperature (*T*_m_) was calculated using a plot of the derivative of the fluorescence intensity against temperature as the *T*_m_. Experiments were conducted in triplicate.

## Supplementary Material



## Data Availability

All data needed to evaluate the conclusions in the paper are present in the paper and/or the Supporting Information. All data sets generated during the current study have been deposited in the Electron Microscopy Data Bank (EMDB) under accession numbers EMD-55303 (CHIMPSELS), EMD-55342, EMD-55341, EMD-55340 (CHIMPSELS S57C C1, I4 and two-penton maps, respectively), and in the Protein Data Bank (PDB) under accession numbers PDB ID: 9SWA (CHIMPSELS) and PDB ID: 9SY5 (CHIMPSELS S57C). Reagents are available from I.B. and C.S. via a material transfer agreement upon request.

## Notes

Additional experimental data of the purification and cryo-EM data processing of the CHIMPSELS ADDomer, structural comparisons of Ad3, CHIMPSELS and CHIMERA ADDomers, negative stain EM images of double cysteine mutant CHIMPSELS and CHIMERA ADDomers, negative stain EM images of ADDomers heated to 45 and 50 °C, purification and cryo-EM data processing of the CHIMPSELS S57C ADDomer, amino acid sequence alignment of PBPs, cryo-EM data collection, and refinement statistics of CHIMPSELS and CHIMPSELS S57C (Figures S1–S10) and (Tables S1–S3) (PDF)

## Abbreviations

ADDomer: Adenovirus dodecahedron-derived nanoparticle
AEBSF: 4-(2-Aminoethyl)­benzenesulfonyl fluoride
AIEX: Anion exchange chromatography
BSA: Bovine serum albumin
CCD: Charge-coupled device detector
CCP-EM: Collaborative Computational Project for Electron Microscopy
COVID-19: Coronavirus disease 2019
Cryo-EM: Cryogenic electron microscopy
CTF: Contrast transfer function
DLS: Dynamic light scattering
DNA: Deoxyribonucleic acid
DSF: Differential scanning fluorimetry
E2EP3: Epitope 3 from Chikungunya virus E2 glycoprotein
FCS: Fourier shell correlation
HPV: Human papillomavirus
kDa: Kilodalton
NaI: Sodium iodide
PBP: Penton base protein
PDB: Protein Data Bank
RGD loop: Arginine-glycine-aspartate loop
RNA: Ribonucleic acid
SARS-CoV-2: Severe acute respiratory syndrome coronavirus 2
SEC: Size exclusion chromatography
SYPRO Orange: Fluorescent dye used in thermal shift assays
TEV: Tobacco etch virus (protease)
Tm: Melting temperature
VLP: Virus-like particle
VL: Variable loop
