# Supplementary material for: Engineering the ADDomer Nanoparticle Vaccine Scaffold for Improved Assembly and Enhanced Stability
Source: ACS Synth Biol. 2026 Mar 17;15(4):1340–52. doi: 10.1021/acssynbio.5c00757 (PMC13097255; doi:10.1021/acssynbio.5c00757)
Supplement: Supplementary file 1 [file sb5c00757_si_001.pdf]

Supporting Information

Engineering the ADDomer Nanoparticle Vaccine Scaffold For Improved Assembly and Enhanced Stability

Authors:

Georgia Balchin[1§], Burak V. Kabasakal[1,2,3 §], Alessandro Strofaldi[4,5], Sophie Hall[1], Charlotte Fletcher[1], Dora Buzas[1], Joshua C. Bufton[1], Sathish K.N. Yadav[1], Dakang Shen[1], Frederic Garzoni[1,6], H. Adrian Bunzel[1,7], Jennifer J. McManus[4], Christiane Schaffitzel[1*], Imre Berger[1,8*]

Affiliations:

[1] School of Biochemistry, University of Bristol, University Walk, Bristol BS8 1TD, UK

[2] Turkish Accelerator and Radiation Laboratory (TARLA), 06830 Ankara, Türkiye

[3] Department of Biological Sciences, Middle East Technical University, 06800 Ankara, Türkiye

[4] School of Physics, University of Bristol, Tyndall Avenue, Bristol, BS8 1TL, UK

[5] Department of Chemistry, Maynooth University, Maynooth, Co. Kildare, Ireland

[6] King's College, Lavington Street, London, SE1 0NZ, UK

[7] Max Planck Institute for Terrestrial Microbiology, 35043 Marburg, Germany

[8] Max Planck Bristol Centre for Minimal Biology, School of Chemistry, University of Bristol, Cantock's Close, Bristol BS8 1TS, UK

[§] contributed equally

* Authors to whom correspondence should be addressed:

Christiane Schaffitzel, email: cb14941@bristol.ac.uk

Imre Berger, email: imre.berger@bristol.ac.uk

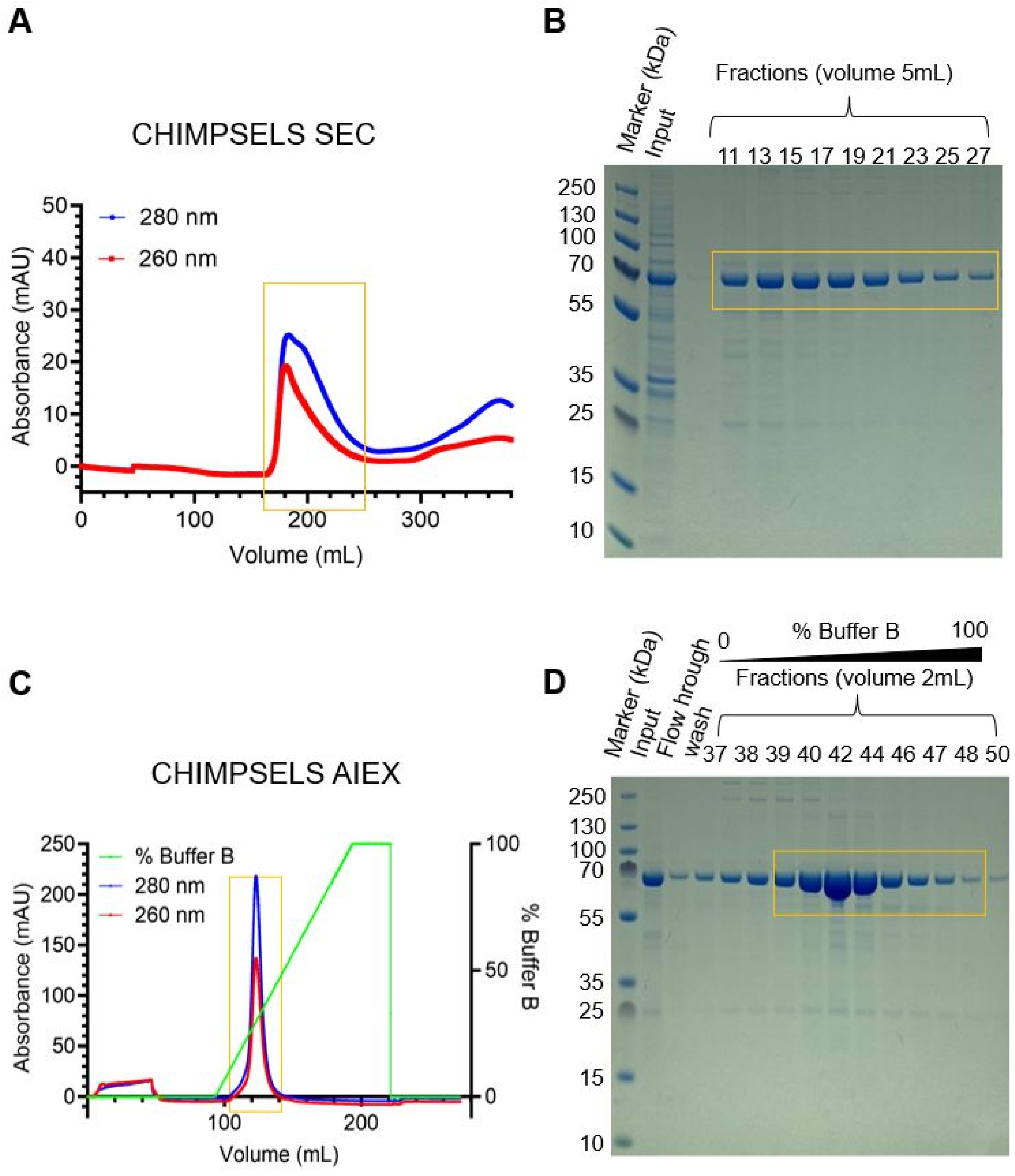

Figure S1: Purification of CHIMPSELS ADDomer (A) Size exclusion chromatography (SEC) of CHIMPSELS ADDomer. Peak fractions (11-27) comprising CHIMPSELS (boxed in yellow) were analysed by SDS-PAGE (B). Fractions comprising CHIMPSELS were pooled, concentrated to 50 mL, and purified by AIEX (C), applying a gradient of 150mM-1M NaCl. Peak fractions (37-50) comprising CHIMPSELS (boxed in yellow) were analysed by SDS-PAGE (D). Fraction volumes are indicated. 280nm and 260nm traces are colored blue and red, respectively.

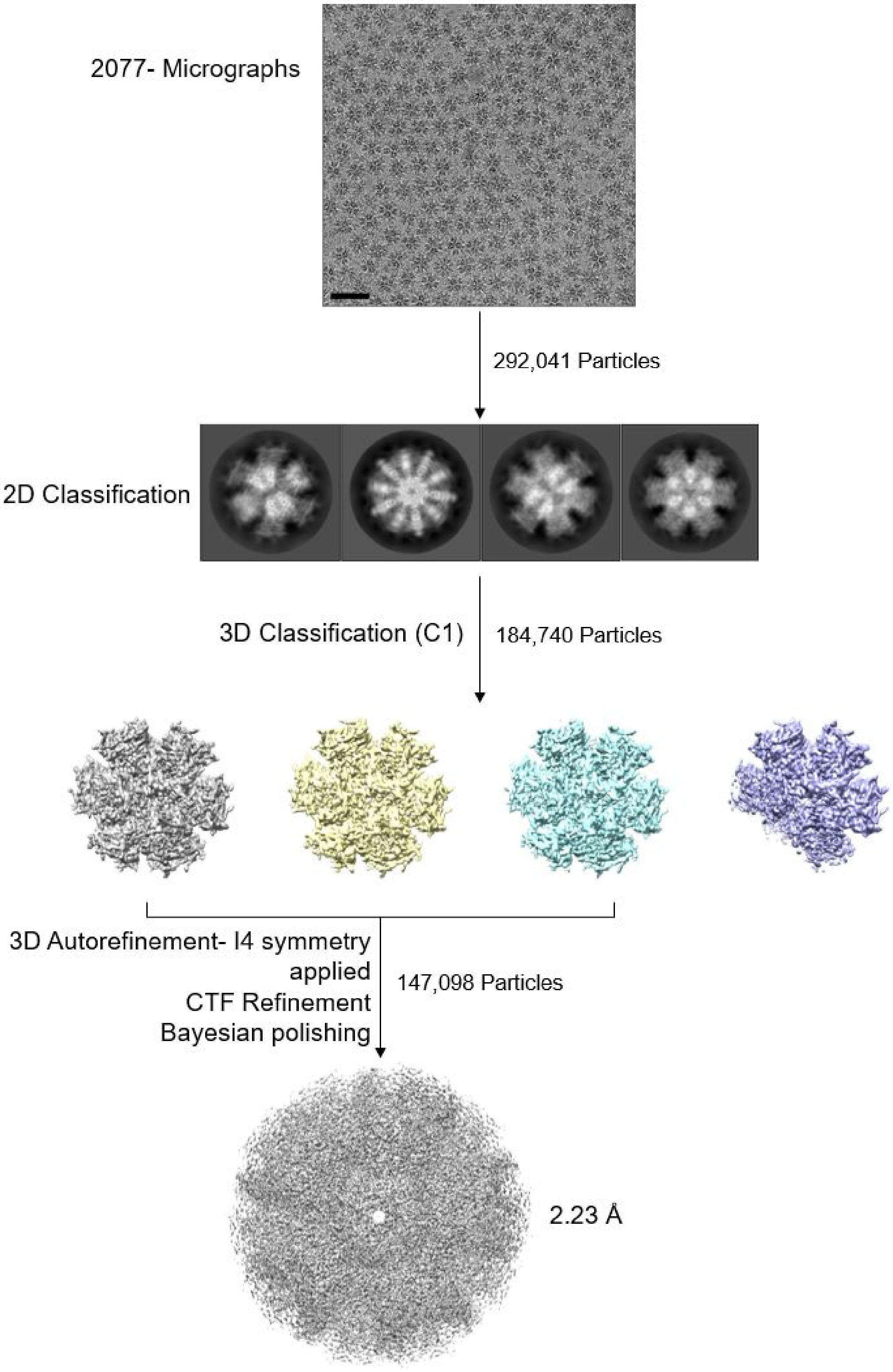

Figure S2: CHIMPSELS cryo-EM workflow. A representative motion corrected EM micrograph (scale bar, 25 nm) is shown at the top, followed by 2D class averages, and cryo-EM maps from 3D classification. Finally, the cryo-EM map is shown (bottom) after 3D autorefinement with I4 symmetry, CTF refinement and Bayesian polishing, resulting in a map at 2.23 Å resolution. Particle numbers used are indicated.

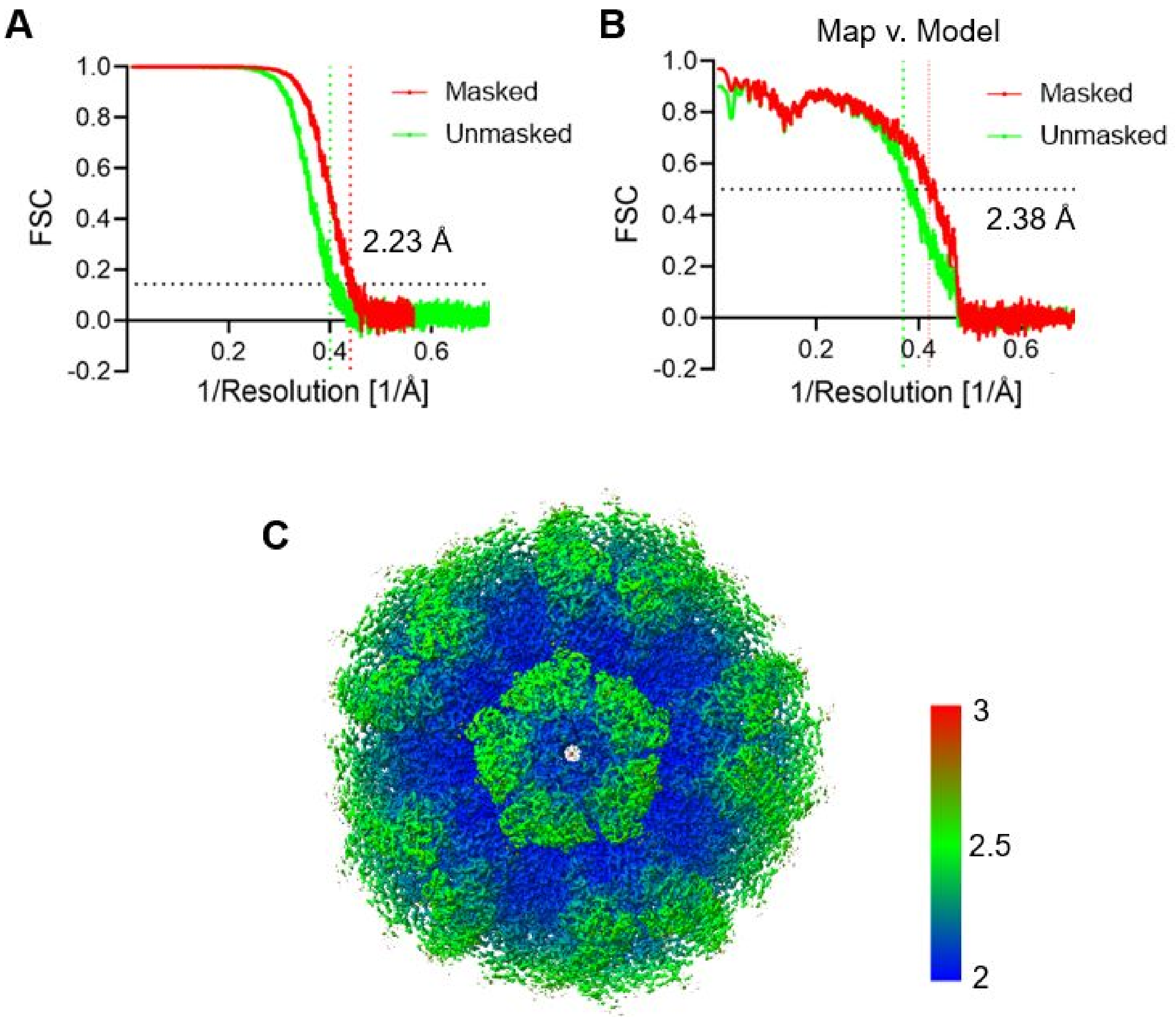

Figure S3: CHIMPSELS FSC curves and local resolution map. (A) Half-map CHIMPSELS FSC curve with the gold standard 0.143 criterion indicating the resolution of 2.23 Å. (B) The model map CHIMPSELS FSC curve calculated between the atomic model and final cryo-EM map. The map model FSC at a 0.5 cutoff reaches a resolution of 2.38 Å. (C) The CHIMPSELS final map showing the local resolution. Color bar indicating resolution (in Å) is shown. The core of the CHIMPSELS is resolved at a resolution of 2 Å while the outer parts of the particle including the flexible VL and RGD are less well defined.

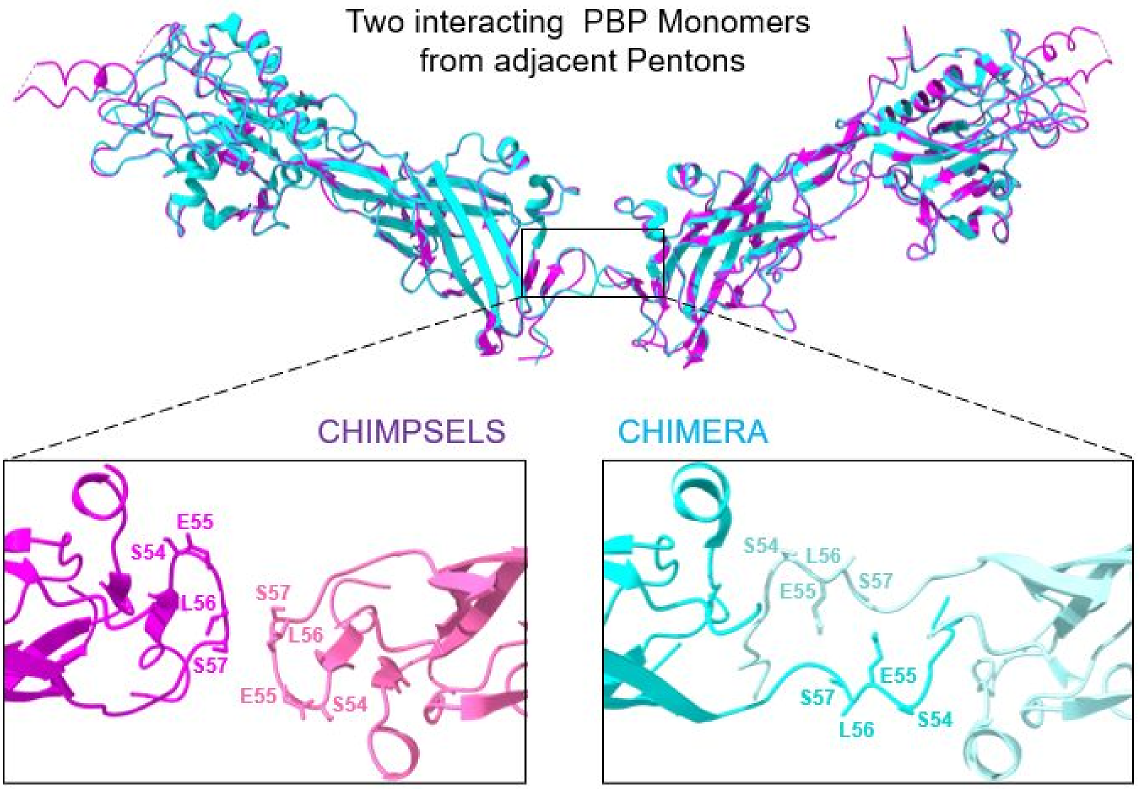

Figure S4: Interactions between PBPs in adjacent pentons in CHIMPSELS and CHIMERA ADDomers by cryo-EM. Two interacting adjacent PBP monomers from CHIMPSELS (magenta) and CHIMERA (cyan) are shown in a ribbon presentation, overlayed to highlight differences in the geometries of the N-termini (top). Zoom-ins show that the CHIMPSELS N-termini adopt a hairpin conformation (left). The CHIMERA structure in contrast is stabilized by a strand-swapped conformation of the N-termini. Residues constituting the SELS tetra-residue motif in both structures are labelled. Residue numbers are indicated.

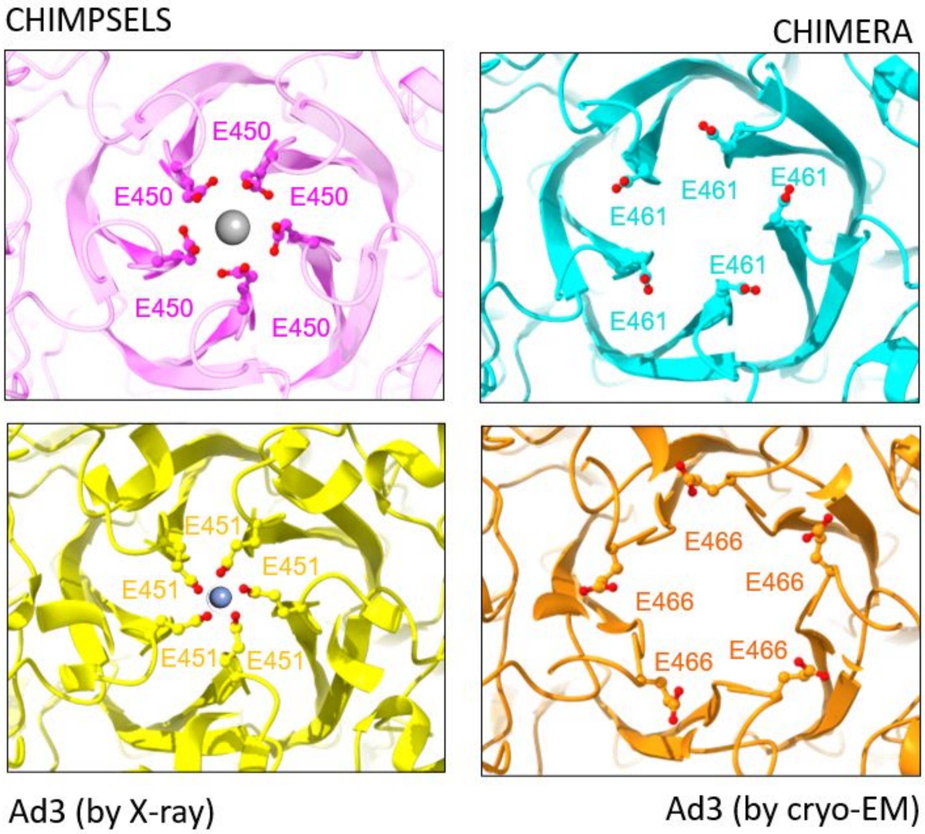

Figure S5: Metal ion coordination in central cavity of ADDomer pentons. In CHIMPSELS, glutamates E450 coordinate a central ion (fray, modelled as potassium). In the X-ray crystal structure of the Ad3 penton (PDB ID: 4ar2), a calcium ion (blue) was identified, tightly coordinated by the glutamates. In both the CHIMERA (PDB ID: 8qbx) and ADDomer Ad3 cryo-EM structures (PDB ID: 6hcr), the central cavity is unoccupied, and the glutamates are rotated away from the central penton axis. Glutamate residue numbers are indicated.

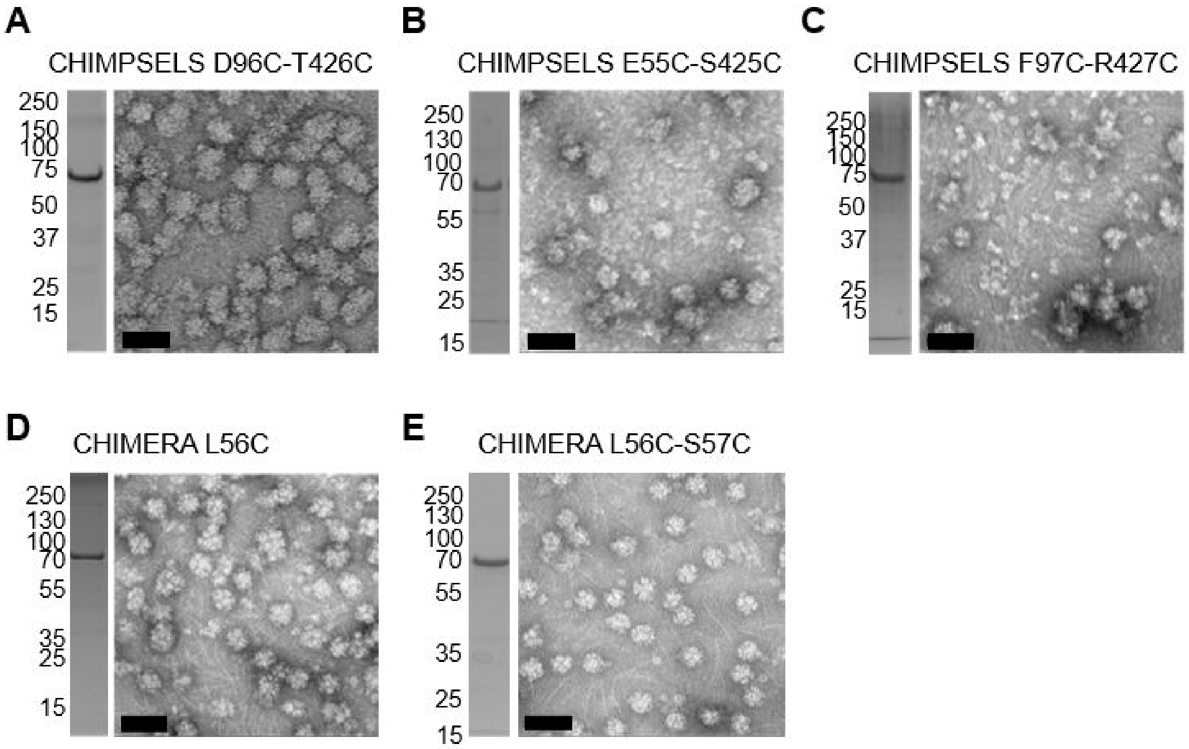

Figure S6: Negative stain EM images of cysteine mutant CHIMPSELS and CHIMERA PBP constructs. Negative stain EM images of CHIMPSELS ADDomer constructs containing double cysteine mutations, evidence deformed, irregular and partly disassembled particles (A-C). Corresponding SDS-PAGE sections indicate highly purified proteins. ADDomers based on the CHIMERA construct comprising one single cysteine mutation (D) and a double cysteine mutation (E) likewise failed to adopt proper dodecahedra. Scale bars (50 nm) are colored in black. Mutations are indicated by residue numbers.

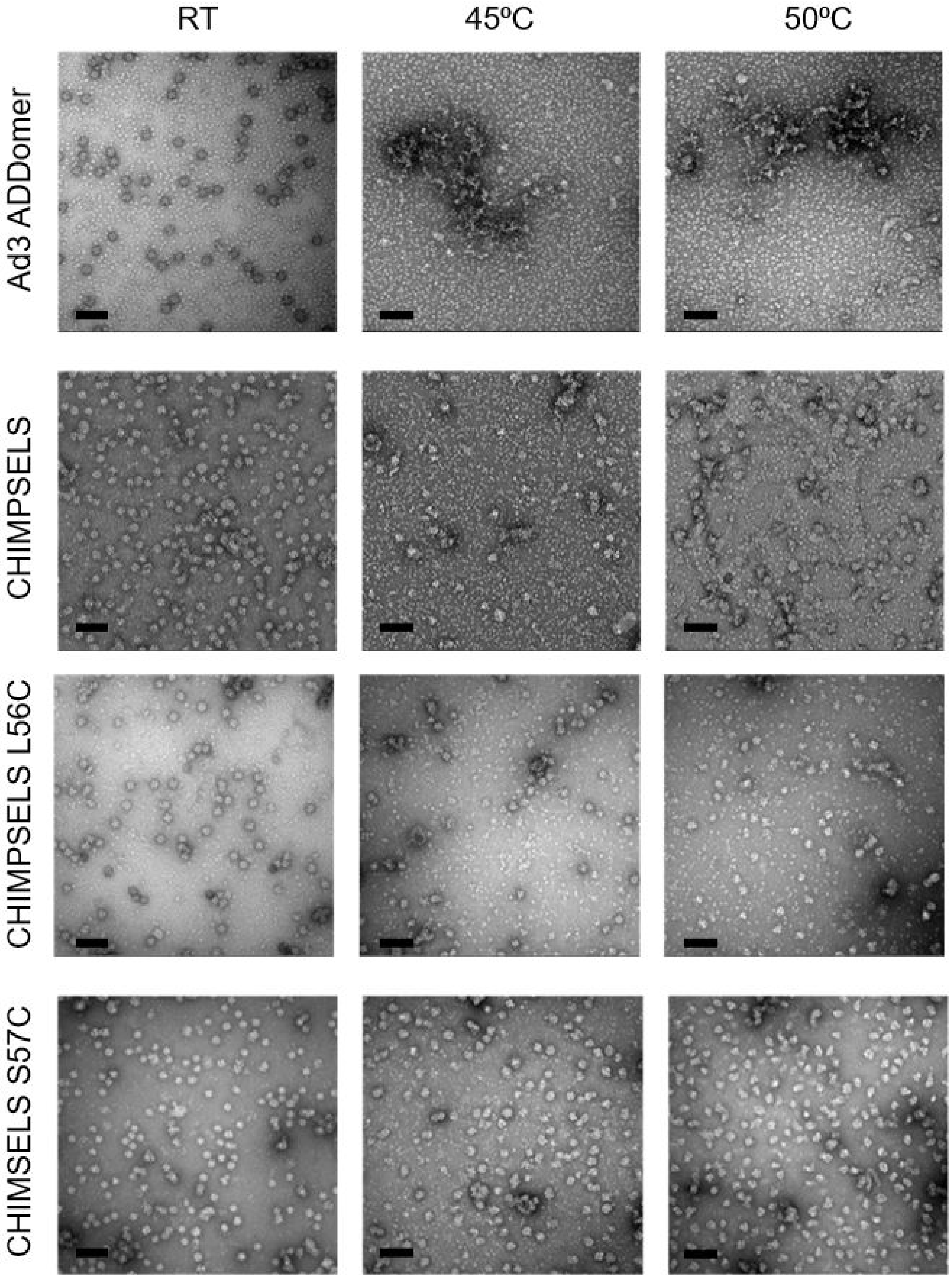

Figure S7: Thermotolerance of ADDomer VLPs. Negative stain EM images of ADDomer constructs stored at room temperature (RT), 45°C and 50°C are shown. Ad3 ADDomer, CHIMPSELS, and CHIMPSELS with single cysteine mutations (L56C, S56C) were analysed. Scale bar (100 nm) is shown in black.

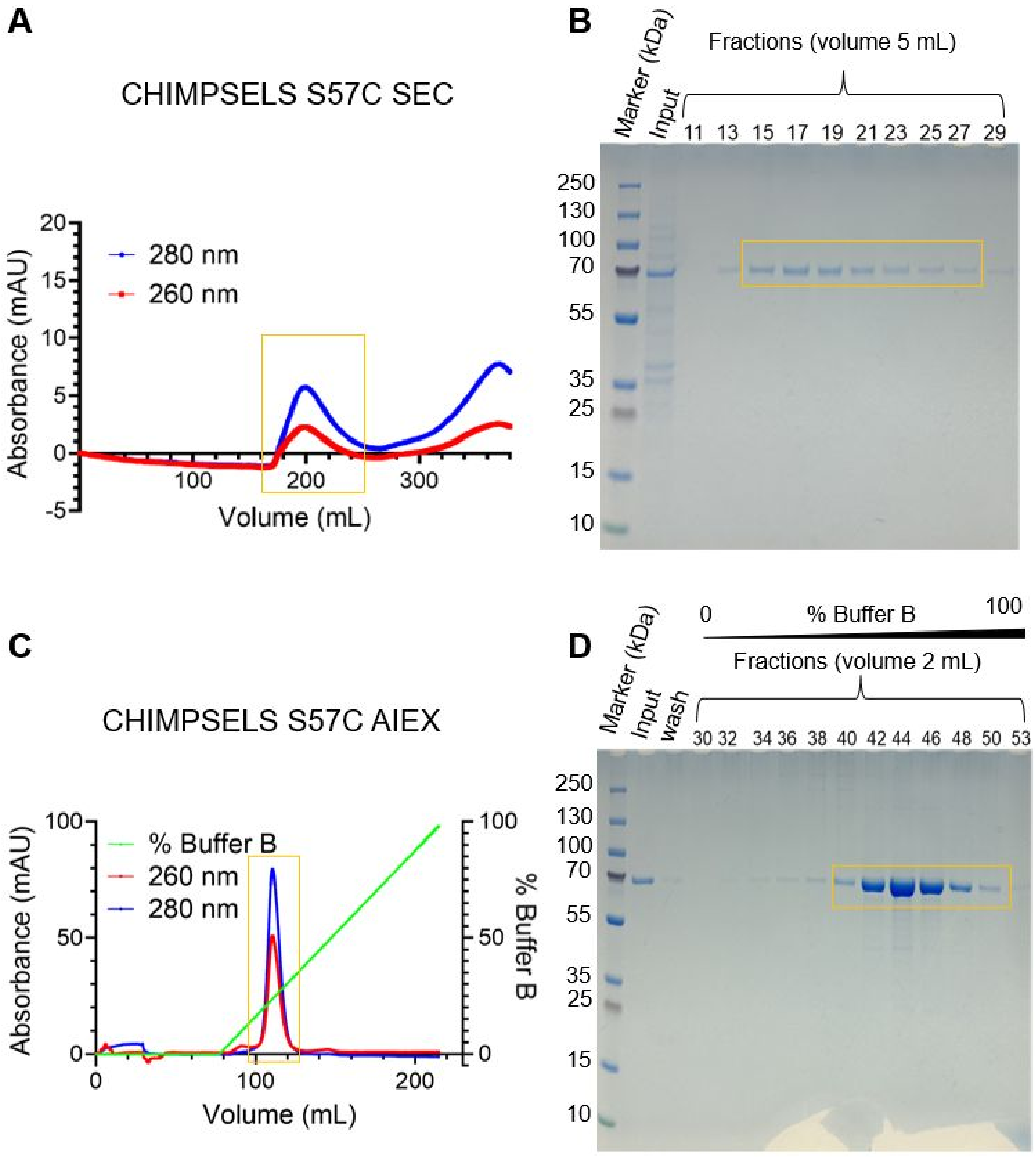

Figure S8: Purification of CHIMPSELS S57C mutant. (A) SEC of CHIMPSELS S57C ADDomer. Peak fractions (11-29) comprising CHIMPSELS S57C (boxed in yellow) were analysed by SDS-PAGE (B). Fractions 15-27 comprising CHIMPSELS S57C protein were pooled, concentrated to 30 mL, and purified by AIEX (C), with a gradient of 150mM-1M NaCl. Peak fractions (40-50) comprising CHIMPSELS S57C (boxed in yellow) were analysed by SDS-PAGE (D). Fraction volumes are indicated. 280nm and 260nm traces are colored blue and red, respectively.

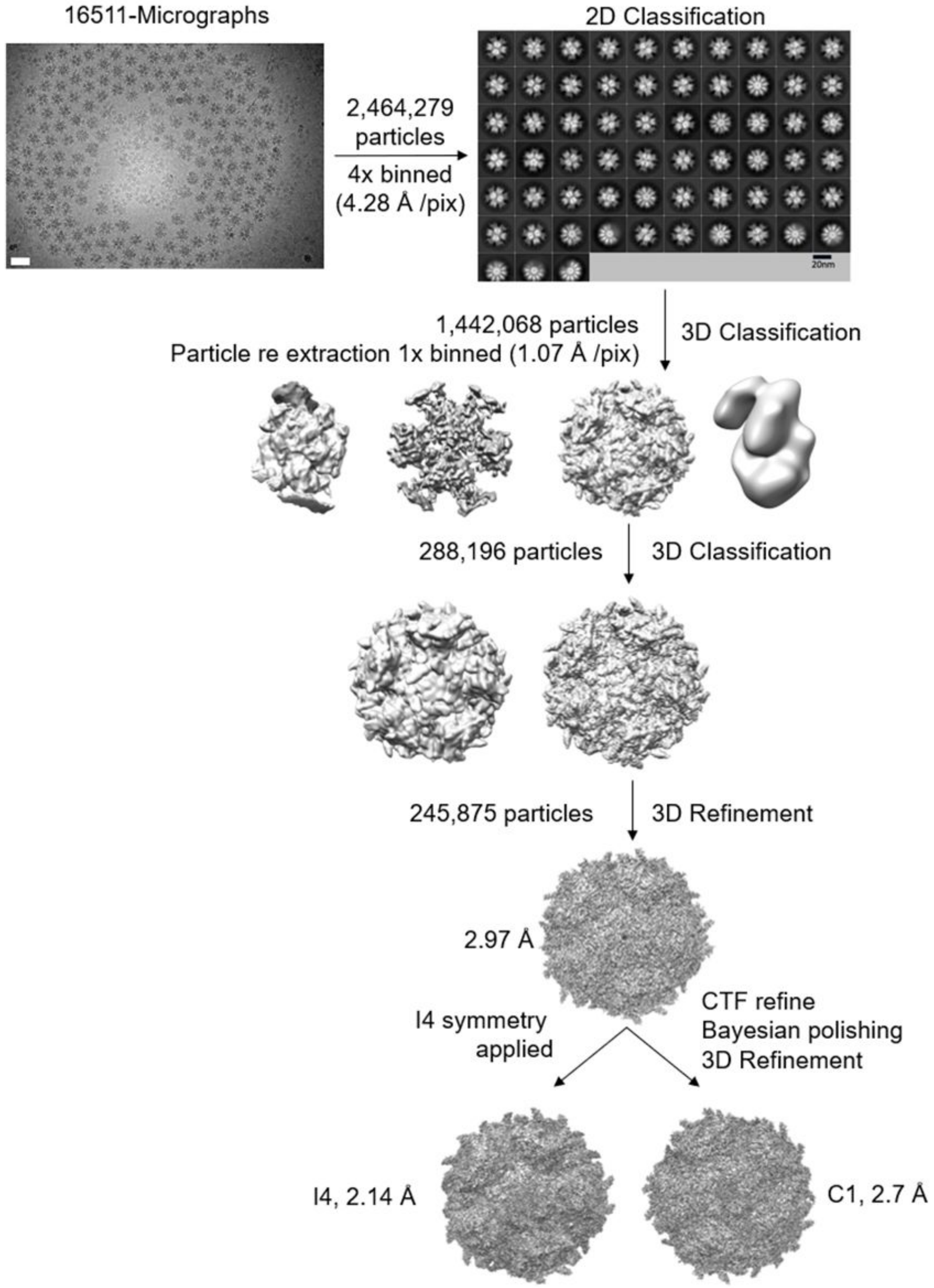

Figure S9: CHIMPSELS S57C cryo-EM workflow. A representative micrograph is shown followed by 2D class averages (scale bar 20 nm). Cryo-EM models are shown following two rounds of 3D classification. The particle was refined to a resolution of 2.97 Å. CTF refinement, Bayesian polishing and 3D refinement was conducted resulting in a map with a resolution of 2.7 Å. I4 symmetry was applied resulting in a map with a resolution of 2.14 Å. Particle numbers are indicated.

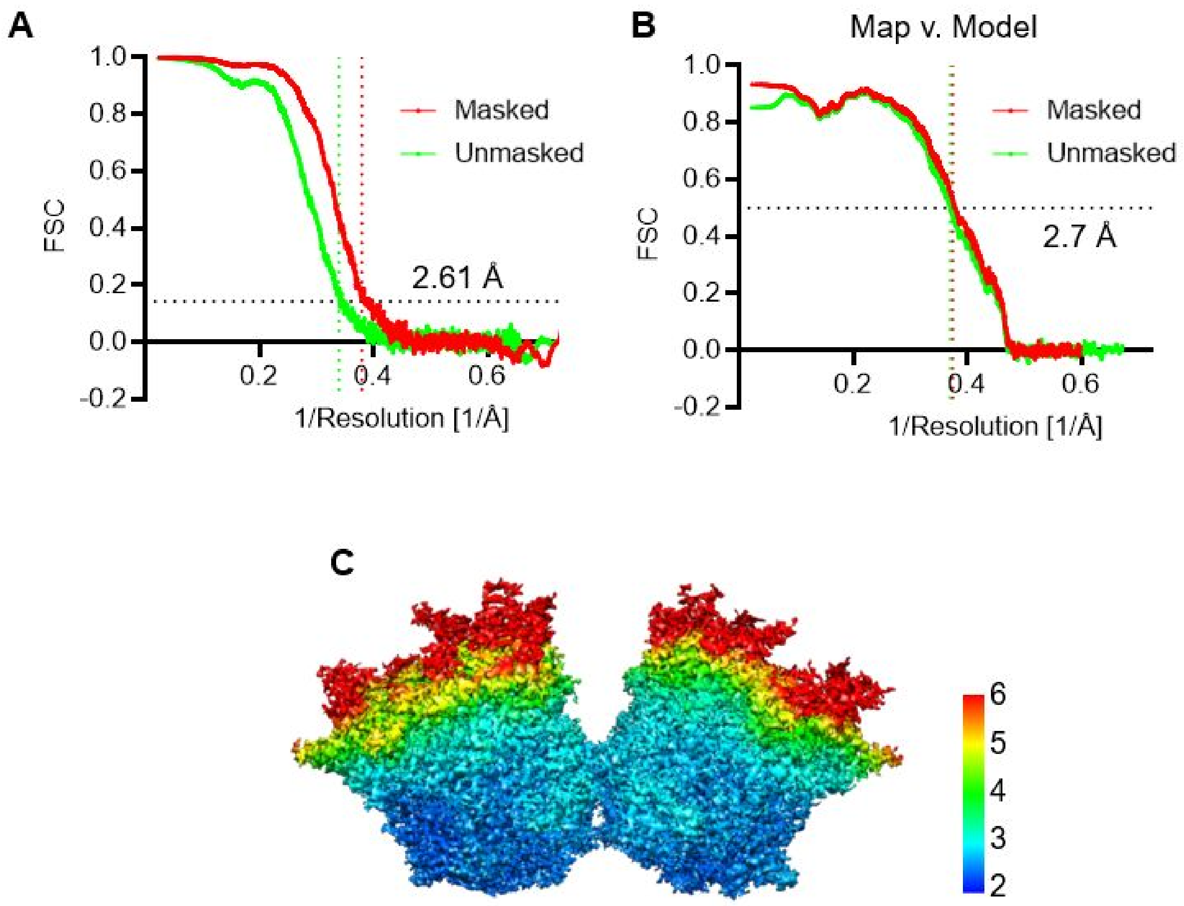

Figure S10: CHIMPSELS S57C FSC curves and local resolution map. (A) Half-map CHIMPSELS S57C FSC curve of two adjacent pentons is shown, with the gold standard 0.143 criterion indicating a resolution of 2.61 Å. (B) Model map CHIMPSELS S57C FSC curve of the two pentons was calculated between atomic model and final cryo-EM map. The map model FSC at a 0.5 cutoff reaches a resolution of 2.7 Å. (C) The CHIMPSELS S57C final two-penton map displaying the local resolution. The core of the CHIMPSELS S57C particle is resolved to 2 Å (blue) whereas the solvent exposed surface regions including the flexible VL and RGD loop exhibit lower resolution (red). Color bar indicating resolution (in Å) is shown.

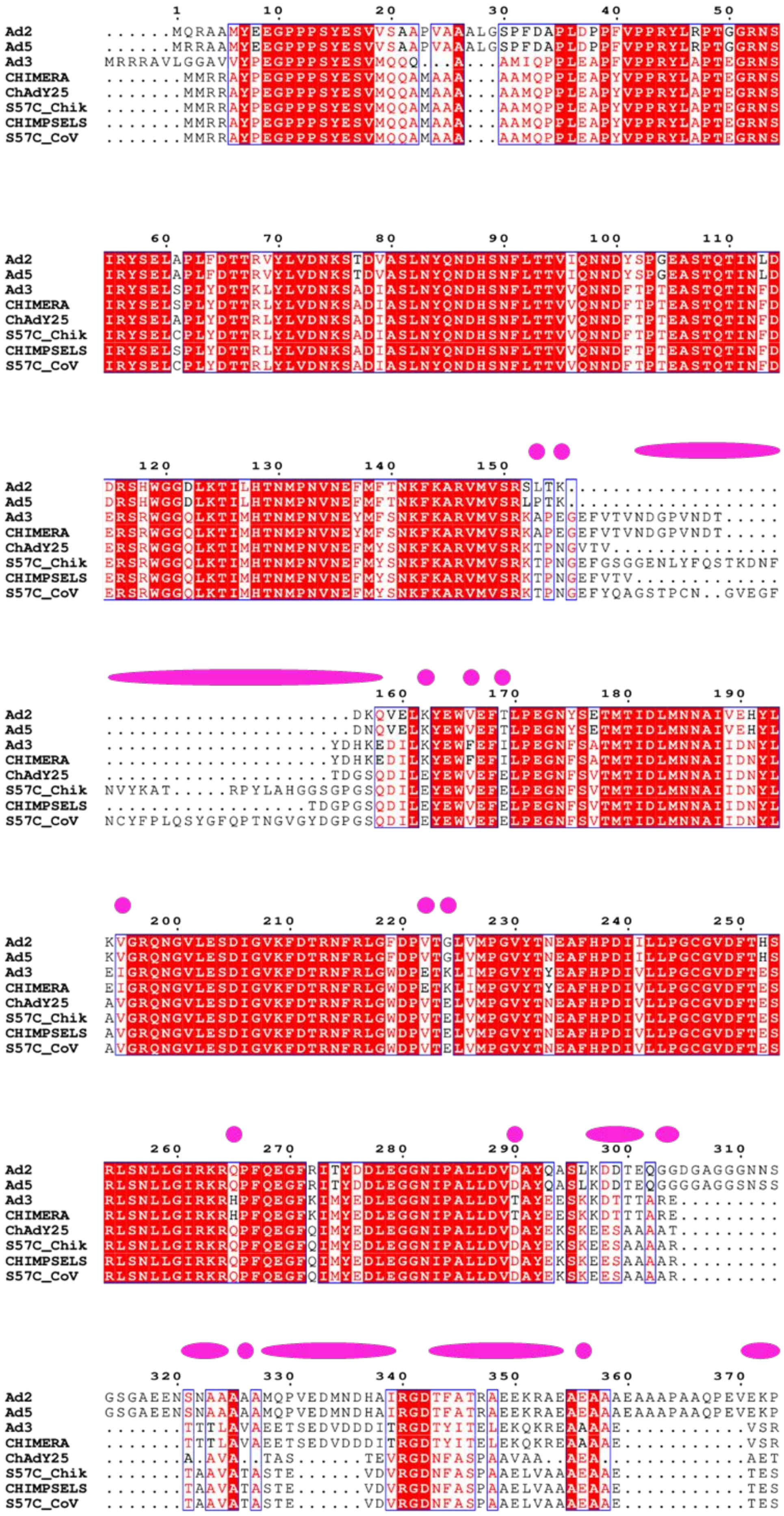

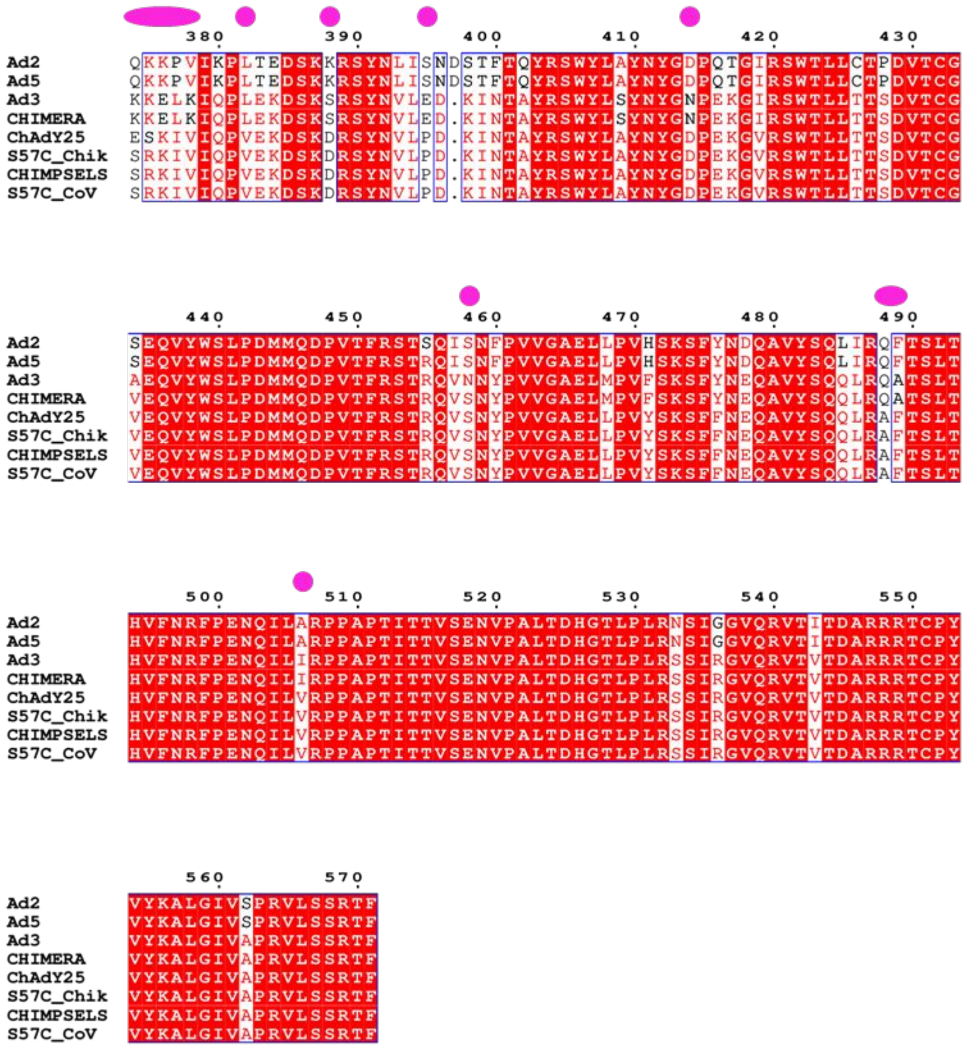

Table S1: Amino acid sequence alignment of full PBPs. The alignment includes penton proteins from the common human adenovirus serotypes Ad2 and Ad5, the chimpanzee serotype ChAdY25 as well as the Ad3, CHIMERA, CHIMPSELS, S57C_Chik and S57C_CoV ADDomer PBPs. Pink circles indicate surface-exposed residues that differ between the CHIMPSELS and Ad3 ADDomer PBPs.

Table S2: Cryo-EM data collection and refinement statistics.

	CHIMPSELS (C1)	CHIMPSELS S57C (C1)
Voltage (V)	200	300
Magnification (nominal)	130 kX	81 kX
Pixel size (Å/pix)	1.05	1.072
Flux (e^{-}/pix./s)	6.065	15.01
Frames per exposure	40	50
Exposure (e^{-}/ Å^2)	44.01	50
Defocus range (µm)	-0.7 to -2.2 (0.5 steps)	-0.8 to -2 (0.4 steps)
Micrographs collected	2920	16511
Particles final	147098	245875
Map sharpening B-factor (Å^2)	74.97	60.7166
Resolution at 0.143 FSC (Å)	2.23	2.71

Refinement

	CHIMPSELS	CHIMPSELS S57C (two pentons)
Composition		
Chains	60	10
Amino acids	26100	4256
Root mean square deviation (RMSD)		
RMSD bonds (Å)	0.006	0.003
RMSD angles (°)	1.757	0.579
Ramachandran values		
Favored (%)	91	96.5
Allowed (%)	9	3.5
Outliers (%)	0	0
Validation		
Rotamer outliers (%)	0.03	3.75
Clash score	18.3	6.4
C-beta outliers (%)	1.45	0.0
CaBLAM outliers (%)	4.30	1.39
CC (mask)	0.78	0.81
MolProbity score	2.28	2.02
Model resolution (Å) 0.5 FSC threshold	2.38	2.7

Table S3: Thermal melting temperatures of ADDomer VLPs.

ADDomer VLP	Thermal melting temperature (T_m) in °C
CHIMPSELS	52.9
CHIMPSELS L56C	53.2
CHIMPSELS S57C	50.2
ADDomer Ad3	54.3
CHIMERA	53.2
CHIMERA S57C	54.2
CHIMPSELS S57C CoV	49.9
CHIMPSELS S57C Chik uncut	51.6
CHIMPSELS S57C Chik cut	53.3
